# Serum Proteomic Profiling by SomaScan Aptamer Array Identifies Candidate Predictive and Monitoring Biomarkers of Distant Recurrence in Triple-Negative Breast Cancer

**DOI:** 10.3390/ijms27209070

**Published:** 2026-10-13

**Authors:** Jina Kim, Sunao Tanaka, Edwardia Fosah, Cynthia Jinno, Hyoyoung Kim, Thanosan Prathifkumar, Yukiko Fukui, Kana Kawanishi, Masahiro Kawashima, Norikazu Masuda, Joshua Levy, Takeo Fujii, Sungyong You, Hideki Furuya

**Affiliations:** 1 Department of Computational Biomedicine, Cedars-Sinai Medical Center, Los Angeles, CA 90069, USA; jina.kim2@csmc.edu (J.K.); joshua.levy@csmc.edu (J.L.); 2 Department of Urology, Cedars-Sinai Medical Center, Los Angeles, CA 90048, USA; hyoyoung.kim@cshs.org; 3 Samuel Oschin Comprehensive Cancer Institute, Cedars-Sinai Medical Center, Los Angeles, CA 90048, USA; sunao.tanaka@csmc.edu (S.T.); cnjinno@gmail.com (C.J.); 4 Department of Electrical and Computer Engineering, The University of Texas at Austin, Austin, TX 78712, USA; edwardiafos@gmail.com; 5 California Institute of Technology, Pasadena, CA 91125, USA; p.thanosan23@gmail.com; 6 Department of Breast Surgery, Graduate School of Medicine, Kyoto University, Kyoto 6068507, Japan; oyuki@kuhp.kyoto-u.ac.jp (Y.F.); kana_k529@kuhp.kyoto-u.ac.jp (K.K.); masa0123@kuhp.kyoto-u.ac.jp (M.K.); masudanori3@kuhp.kyoto-u.ac.jp (N.M.); 7 Pathology and Laboratory Medicine, Cedars-Sinai Medical Center, Los Angeles, CA 90048, USA; 8 Women’s Malignancies Branch, Center for Cancer Research, National Cancer Institute, National Institutes of Health, Bethesda, MD 20892, USA; takeo.fujii@nih.gov; 9 Department of Biomedical Science, Cedars-Sinai Medical Center, Los Angeles, CA 90048, USA

**Keywords:** triple-negative breast cancer, serum proteomics, SomaScan, metastasis, recurrence, biomarker, liquid biopsy

## Abstract

Triple-negative breast cancer (TNBC) lacks a blood-based biomarker for predicting or monitoring distant recurrence. We profiled 39 serum samples from 38 women (10 healthy controls; 12 non-recurrent [NR]; eight diagnosis-timepoint samples from patients who later recurred [DR-DIG]; nine recurrence-timepoint samples [DR-REC]) using the SomaScan v5.0 aptamer platform). Public tumor-tissue transcriptomic cohorts were used for supportive and exploratory analyses. No protein reached a false discovery rate (FDR) < 0.05 for NR vs. DR-DIG; granzyme A (GZMA) and endoplasmic reticulum aminopeptidase 1 (ERAP1) showed the strongest nominal signals. For DR-DIG vs. DR-REC, 38 aptamers (37 proteins) reached FDR < 0.05, enriched for RNA-binding and nuclear-envelope proteins; this signature was retained in leave-one-out and storage-adjusted analyses. Higher GZMA mRNA was associated with longer recurrence-free survival in two independent cohorts (hazard ratio [HR] = 0.795, *p* = 0.018; HR = 0.186, *p* = 0.0057). Monitor genes showed near-chance concordance with metastatic tissue, and the source of the signature could not be resolved. An exploratory four-gene elastic-net panel reached a concordance index of 0.613 internally but 0.559 externally. GZMA is a candidate serum biomarker of TNBC recurrence risk, and the monitor signature is a preliminary observation. Both require protein-level validation in independent, prospectively collected serum cohorts.

## 1. Introduction

Triple-negative breast cancer (TNBC), which is defined by the absence of estrogen receptor, progesterone receptor, and human epidermal growth factor receptor 2 (HER2) expression, accounts for approximately 15% of breast cancer diagnoses but a disproportionate share of breast cancer mortality, driven largely by a high rate of early distant recurrence [1]. Despite recent advances, including antibody–drug conjugates such as trastuzumab deruxtecan [2] and sacituzumab govitecan [3], TNBC still lacks broadly effective targeted therapies comparable to endocrine therapy for hormone receptor-positive disease or HER2-directed therapy for HER2-positive disease. Consequently, systemic treatment for most patients continues to rely heavily on chemotherapy, with limited options for preventing metastatic recurrence. Distant recurrence in TNBC commonly occurs within the first three to five years after diagnosis. Once distant metastasis becomes clinically and radiographically detectable, the disease is generally incurable with poor survival outcomes [4]. This devastating clinical course highlights the urgent need to develop biomarkers that can identify patients at high risk of distant organ metastasis. Tissue-based biomarkers have been investigated extensively and provide valuable biological information. However, tissue collection requires an invasive biopsy or surgery and captures only a single spatial and temporal snapshot of a highly heterogeneous and evolving tumor, which limits clinical utility for recurrence prediction and longitudinal surveillance. Moreover, after resection of the primary breast tumor, repeated tissue sampling is generally not feasible for monitoring the emergence and evolution of occult micrometastatic disease. These limitations make tissue-based biomarkers poorly suited for longitudinal assessment of metastatic risk and early detection of recurrence. Blood-based assays offer a minimally invasive and repeatable alternative that could overcome these limitations. However, there remain two major unmet needs. First, there is currently no clinically validated blood-based assay capable of identifying patients with a newly diagnosed TNBC at high risk of future distant recurrence at the time of diagnosis, when treatment intensification decisions are made. Second, there is no blood-based assay that can detect the earliest molecular signal of emerging metastatic recurrence after completion of curative-intent multimodal therapy before metastatic recurrence becomes clinically or radiographically evident. Addressing these unmet needs requires circulating, and ideally secreted, protein-based biomarkers that can monitor recurrence risk longitudinally and reflect the evolving biology of micrometastatic disease.

Recent advances in aptamer-based proteomic platforms enable simultaneous quantification of several thousand serum or plasma proteins from a single small-volume sample. The SomaScan platform (SomaLogic/Standard BioTools), in its v5.0 configuration, measures 10,551 human protein targets and has been applied across a range of oncology and cardiometabolic discovery contexts [5,6]. Serum proteomic discovery by this method offers a key advantage over transcriptomic profiling of primary tumor tissue: it directly measures proteins, the analyte class most plausibly translatable into clinically deployable blood-based assays, providing a more direct path from biomarker discovery to clinical implementation.

These two prior gaps motivated a proteomics-first analytic strategy for this study. First, transcriptomic discovery in primary tumor tissue cannot, by construction, capture proteins that enter the bloodstream after the primary tumor has been resected—a limitation directly relevant to monitoring of distant recurrence, where the analyte of interest may originate from micrometastatic deposits or from the microenvironment of the seeding sites rather than the excised primary tumor. Second, serum protein abundance reflects the integrated effects of gene transcription, translation, secretion, and cell turnover, all of which contribute to the signal for metastatic recurrence. Restricting discovery to transcriptomically nominated candidates might lead to missing signals from these aspects. Based upon this rationale, we first performed unbiased serum proteomic profiling using SomaScan to identify biomarker candidates associated with distant recurrence and then used independent public transcriptomic cohorts to test whether the corresponding proteins exhibited concordant expression patterns in primary tumors or metastatic tissues and to assess their associations with clinical outcomes.

To address these questions, we analyzed serum samples from patients with TNBC enrolled in the BORN-Biobank using two complementary comparisons. The prediction comparison asks whether serum obtained at diagnosis differs between patients who never recur (NR) and those who subsequently developed distant recurrence (DR-DIG)—i.e., whether recurrence risk is legible in serum before clinical recurrence. The monitor comparison asks whether serum obtained at the time of diagnosis (DR-DIG) differs from serum obtained at the time of clinically detectable recurrence (DR-REC)—i.e., whether metastatic disease leaves a detectable serum protein signature at the moment it becomes clinically evident.

Because differential serum protein abundance can arise from several distinct biological processes—increased tumor secretion, altered vascular permeability, or release from dying or turning-over cells—we further asked whether the resulting proteins were annotated as components of the classical secretome and what biological processes they were enriched for before proposing any mechanistic interpretation. We then sought supportive evidence at the tumor-tissue transcript level for the two leading candidate findings—the prediction hit granzyme A (GZMA) and the monitor-model gene set—in public Gene Expression Omnibus (GEO) transcriptomic cohorts with matched clinical outcomes or matched primary-versus-metastatic tissue pairs and tested whether a small composite panel built from the serum-nominated candidates would generalize to an independent validation cohort. We report these results, including a negative composite-panel external validation result, as preliminary data supporting a subsequent, adequately powered prospective study.

## 2. Results

This study comprised three tiers of analysis, which we distinguish throughout. First is serum discovery: all candidate proteins (GZMA, ERAP1, and the 38 monitor-model aptamers) were identified from the SomaScan serum data (Section 2.1, Section 2.2, Section 2.3, Section 2.4 and Section 2.5), and no candidate was nominated from public data. Second is exploratory panel modeling: because the serum cohort has no time-to-event endpoint, a composite panel built from the serum-nominated prediction candidates was fitted and externally tested in public tumor-tissue transcriptomic cohorts (Section 2.6). Third are supportive tissue-transcriptomic analyses: public GEO cohorts were used to ask whether the genes encoding the serum candidates are associated with recurrence or metastasis at the tumor-tissue mRNA level (Section 2.7). Because these analyses test a different analyte (mRNA) in a different compartment (tumor tissue), they provide supportive evidence and do not validate the serum protein findings.

### 2.1. SomaScan Serum Proteomic Cohort and Quality Control

**Figure 1:**
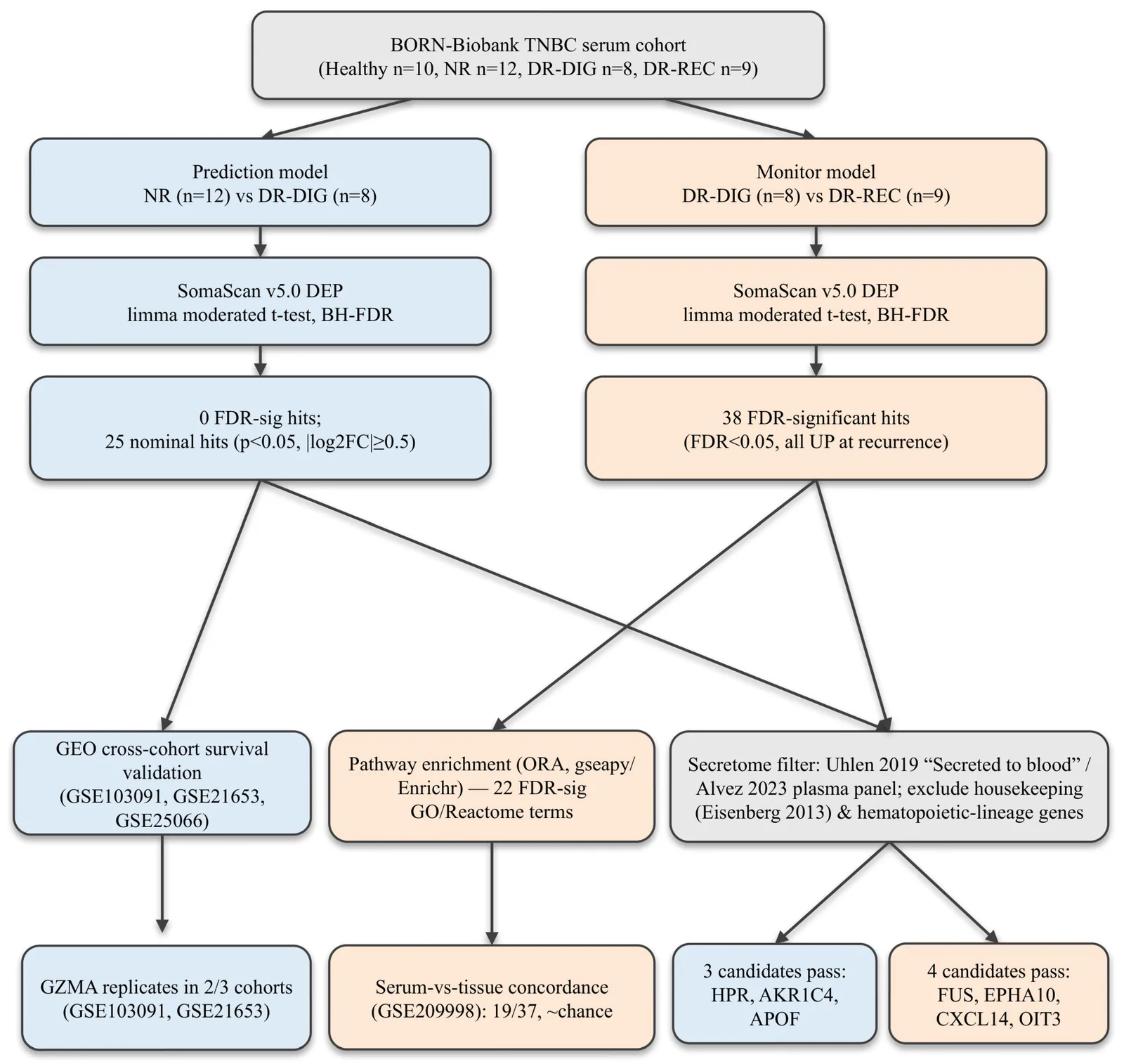
Workflow of the SomaScan proteomic discovery and validation pipeline. Blue: prediction-model branch (no recurrence [NR] vs. diagnosis-timepoint serum from patients who later recurred [DR-DIG]). Orange: monitor-model branch (DR-DIG vs. serum at clinically apparent recurrence [DR-REC]). Grey: steps shared by both models. **Secretome filtering used the HPA secretome classification [7], the HPA pan-cancer blood-proteome panel [8], and the Eisenberg and Levanon housekeeping-gene list [9].** DEP: differential expression protein analysis; BH-FDR: Benjamini–Hochberg false discovery rate; ORA: over-representation analysis; GEO: Gene Expression Omnibus.

Thirty-nine serum samples from 38 women (10 healthy controls and 28 patients with TNBC) with biobanked longitudinal follow-up (BORN-Biobank, Kyoto University) were profiled on the SomaScan v5.0 aptamer platform (ANML-normalized relative fluorescence units, log_2_-transformed; of the 11,083 aptamers on the panel, the 10,551 human aptamers passing platform-level quality control [ColCheck = PASS] were analyzed). Four sample groups were defined: healthy controls (n = 10), non-recurrent TNBC patients sampled at a comparable timepoint (NR, n = 12), diagnosis-timepoint serum from patients who later developed distant recurrence (DR-DIG, n = 8), and recurrence-timepoint serum from patients with clinically apparent recurrence (DR-REC, n = 9). Note that DR-DIG and DR-REC are largely non-overlapping patient sets, not paired longitudinal samples: only one patient contributed both a diagnosis-timepoint and a recurrence-timepoint sample to the analyzed groups. A second potential pair exists in the raw biobank delivery, but the sample collected at diagnosis was excluded for quality-control failure, leaving the sample collected at recurrence as an unpaired recurrence-only sample. The monitor comparison should accordingly be interpreted as a between-group (largely between-patient) comparison; see Discussion for the implications of this design feature for interpreting the resulting signature. Four DR-REC samples represented de novo American Joint Committee on Cancer (AJCC) Stage IV disease (metastatic at presentation, i.e., no true “recurrence” interval) rather than true distant recurrence after a disease-free interval; these were retained in the primary DR-REC group with an explicit sensitivity analysis excluding them. All comparisons reported here are biological replicates (one serum sample per patient per timepoint); no technical replicates were pooled. The overall study design and sample groups are summarized in Figure 1. Cohort demographics and quality-control metrics are summarized in Table 1.

### 2.2. Prediction Model (NR vs. DR-DIG): No FDR-Significant Hits, Nominal Signal Led by ERAP1 and GZMA

**Figure 2:**
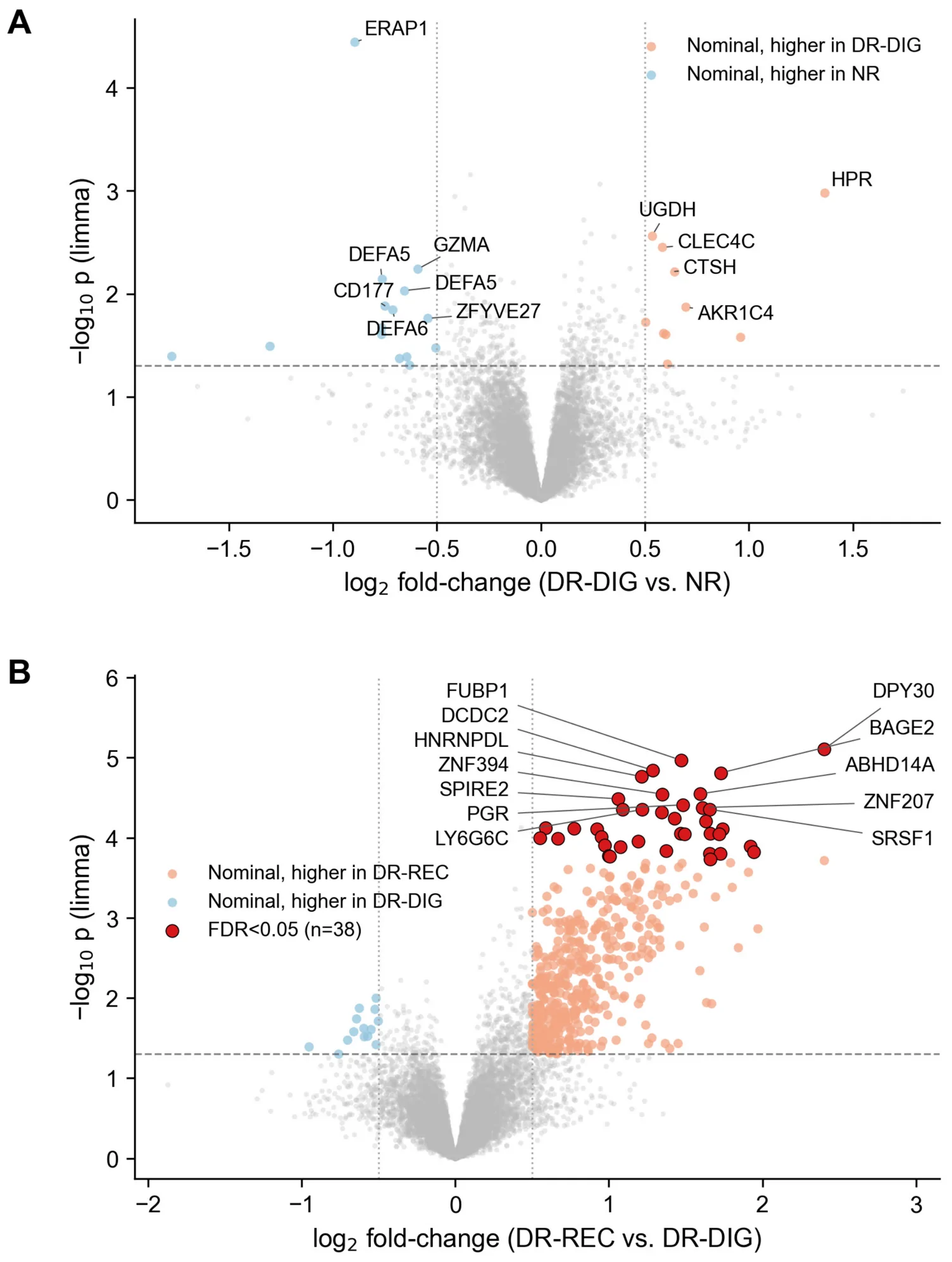
Differential expression protein (DEP) volcano plots. (**A**) Prediction model (no recurrence [NR] vs. DR-DIG), 0 FDR-significant aptamers; 25 aptamers meeting the nominal discovery threshold (*p* < 0.05, |log_2_ fold-change| ≥ 0.5). (**B**) Monitor model (diagnosis-timepoint serum from patients who later recurred [DR-DIG] vs. serum at clinically apparent recurrence [DR-REC]); 38 aptamers reaching Benjamini–Hochberg false discovery rate (FDR) < 0.05 (limma moderated *t*-test, primary test).

We first tested whether diagnosis-timepoint serum protein abundance differs between patients who will and will not later recur (Prediction model). The prediction comparison (NR, n = 12, vs. DR-DIG, n = 8) yielded zero FDR-significant proteins under the moderated *t*-test (limma, BH-FDR; the primary test, selected because Welch’s *t*-test yielded unstable per-gene variance estimates and the Mann–Whitney U test [MWU] produced discrete rank-tie artifacts at this sample size; see Methods), Welch’s *t*-test, or MWU. This null result is consistent with the comparison being underpowered at n = 12 vs. 8 rather than indicating an absence of biological signal. Twenty-five aptamers (24 unique genes) reached the pre-specified nominal discovery threshold (limma *p* < 0.05 AND |log_2_ fold-change [log_2_FC]| ≥ 0.5). The top nominal hits were ERAP1 (log_2_FC = −0.90, up in NR, *p* = 3.6 × 10^−5^), GZMA (log_2_FC = −0.59, upregulated [up] in NR, *p* = 0.0057), KLRG2 (log_2_FC = −1.78, up in NR, *p* = 0.0403), haptoglobin-related protein (HPR; log_2_FC = +1.36, up in DR-DIG, *p* = 0.0010), and UDP-glucose 6-dehydrogenase (UGDH; log_2_FC = +0.54, up in DR-DIG, *p* = 0.0027). Both ERAP1 and GZMA were higher in patients who did not recur, a pattern that may reflect an immune-surveillance state (ERAP1: major histocompatibility complex class I [MHC-I] antigen presentation; GZMA: cytotoxic T-lymphocyte/natural killer-cell effector granzyme) present at diagnosis in patients whose disease did not go on to recur. The full volcano plot and complete 25-aptamer nominal hit list are shown in Figure 2A and Table 2, respectively.

### 2.3. Monitor Model (DR-DIG vs. DR-REC): 38 FDR-Significant Hits (37 Unique Genes), Retained After Exclusion of De Novo Stage IV Samples

We next tested whether serum protein abundance differs between the diagnosis timepoint and the timepoint of clinically apparent recurrence (Monitor model). The monitor comparison (DR-DIG, n = 8, vs. DR-REC, n = 9) yielded 38 FDR-significant aptamer hits (37 unique gene symbols; one gene, heterogeneous nuclear ribonucleoprotein A1 [HNRNPA1], represented by two aptamers) under the primary limma test (FDR range 0.036–0.050), all directionally higher in the DR-REC group. This is a substantially larger and more stable hit set than obtained under MWU (81 FDR hits, subject to rank-tie granularity at this sample size) or Welch’s *t*-test (0 FDR hits, unstable per-gene variance estimates at n = 8–9); a total of 33 of the 38 limma hits (87%) overlap the original MWU-81 set (Figure 2B, Table 3). A sensitivity analysis excluding the four de novo Stage IV samples (true recurrence-only comparison, n = 5 vs. 8) found all 38 hits nominally significant, with 15 of 38 (39%) showing perfect rank separation (all true-recurrence samples exceeding all diagnosis-timepoint samples) (Supplementary Table S1). All 38 hits also remained higher in DR-REC with *p* < 0.05 when each DR-REC sample was left out in turn and after adjustment for serum storage duration (Supplementary Tables S3 and S4). These data suggest the monitor signal is not explained by the inclusion of de novo Stage IV disease in the DR-REC group.

Representative monitor hits, illustrating the functional categories detailed below, included DPY30 (log_2_FC = +2.40, histone H3K4 methyltransferase complex component), far upstream binding protein 1 (FUBP1, log_2_FC = +1.47), doublecortin domain-containing protein 2 (DCDC2, log_2_FC = +1.29), lamin B2 (LMNB2, log_2_FC = +1.94), thymopoietin (TMPO, log_2_FC = +1.92), and the FET-family RNA-binding proteins Ewing sarcoma breakpoint region 1 (EWSR1, log_2_FC = +1.47) and fused in sarcoma (FUS, log_2_FC = +1.66). Descriptively, this hit set is dominated by RNA-binding/nuclear proteins and chromatin-associated factors; we examined this characterization by enrichment analysis below.

### 2.4. Exploratory Secretome Annotation of DEP Hits

We next asked whether the 38 monitor hits and the 25 prediction nominal hits were annotated as components of the classical secretome or the measured cancer plasma proteome, applying the lab’s standard filter logic against the actual primary reference sources: Human Protein Atlas (HPA) human secretome classification (Data File S2: 2640 genes, of which 730 are annotated as secretome) for secretome status [7] and HPA pan-cancer blood-proteome profiling dataset (1463 proteins measured across 12 cancer types by proximity extension assay, BioStudies accession S-BSST935) for cancer plasma-proteome [8]; Eisenberg and Levanon’s housekeeping gene list and HPA-annotated hematopoietic/blood-lineage-restricted genes (Human housekeeping genes) were excluded as before [9].

Of the 38 monitor hits, two were annotated as secreted to blood in the HPA secretome classification, and four were present in the HPA pan-cancer blood-proteome panel (union: 5/38, 13%); a total of 15 (39%) were excluded as Eisenberg housekeeping genes, and three (8%) were excluded as hematopoietic lineage restricted. After all exclusions, four of 38 aptamers (four unique genes) passed the full filter: fused in sarcoma (FUS), EPH receptor A10 (EPHA10), C-X-C motif chemokine ligand 14 (CXCL14), and oncoprotein-induced transcript 3 (OIT3) (Table 4). Of the 25 prediction nominal hits, three aptamers (three unique genes) passed: haptoglobin-related protein (HPR), apolipoprotein F (APOF), and aldo-keto reductase family 1 member C4 (AKR1C4) (Table 4). That only four of 38 (11%) monitor hits pass this filter while the majority of these same genes’ canonical biology is nuclear/intracellular suggests that most of these proteins reach serum by routes other than classical secretion (e.g., release during cell death or turnover), although these annotations do not identify the cellular source: most of the 38-protein signature is neither predicted-secreted nor empirically detected in an independent cancer blood-proteome survey. FUS illustrates this point: it is measurable in cancer patient blood [8] despite carrying no predicted secretory signal (absent from the entire secretome candidate list, not merely mis-classified as non-secreted) and is consistent with the FUS serum-versus-tissue discordance noted below.

### 2.5. Exploratory Pathway Enrichment Analysis of the 38 Monitor Hits

**Figure 3:**
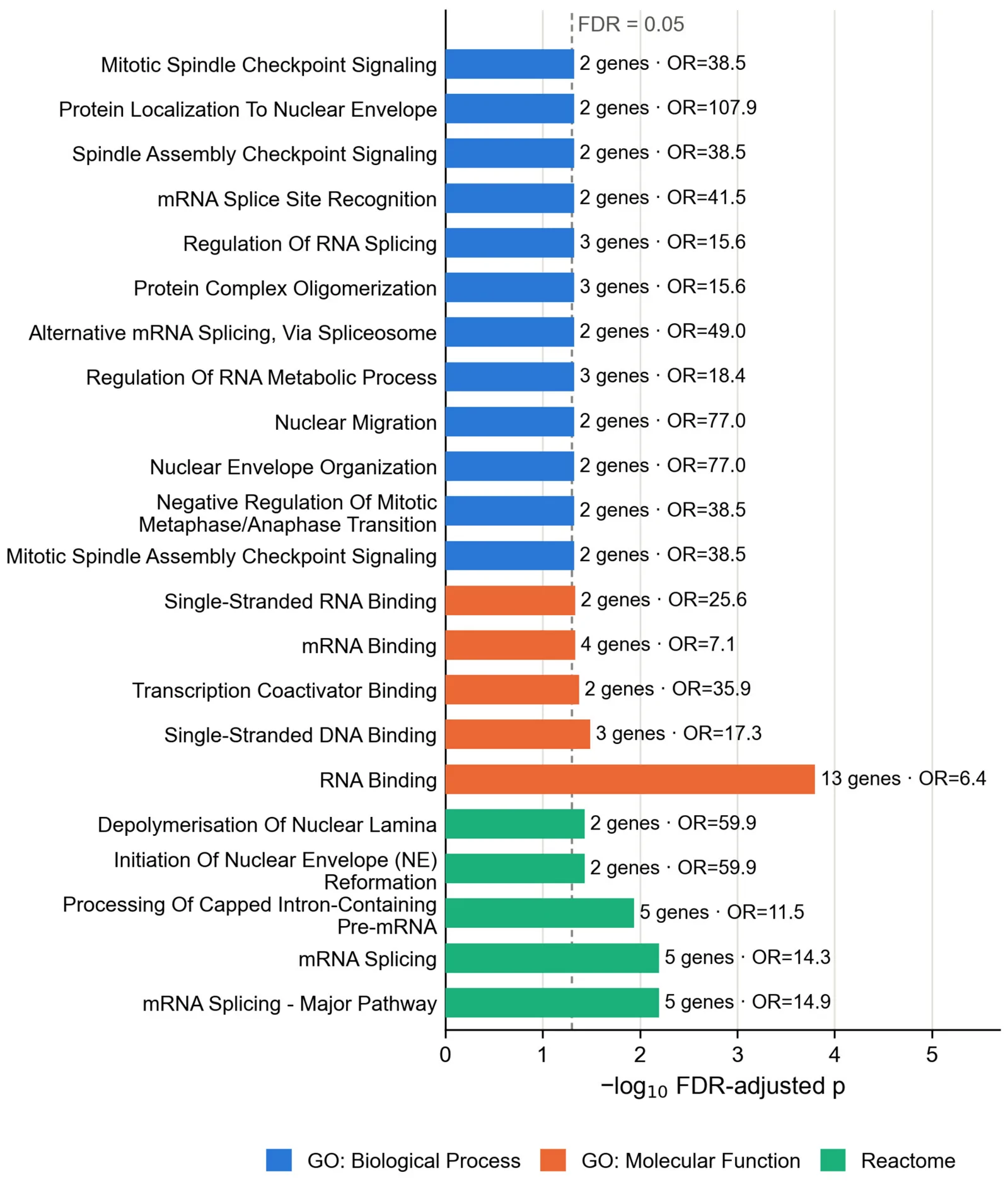
Over-representation analysis (ORA; gseapy/Enrichr, Fisher’s exact test, Benjamini–Hochberg false discovery rate [FDR]) of the 37 unique monitor-model hit gene symbols, tested against a background restricted to the 9482 genes measured on the SomaScan v5.0 panel. All 22 Gene Ontology (GO) Biological Process, GO Molecular Function, and Reactome terms reaching FDR < 0.05 are shown, ranked by odds ratio and labeled with overlap gene count.

To move beyond descriptive characterization, we performed over-representation analysis (ORA; gseapy/Enrichr, Fisher’s exact test, BH-FDR) on the 37 unique monitor-hit gene symbols against a background restricted to the 9482 unique genes measured on the SomaScan v5.0 panel itself (rather than a whole-genome background, which would bias toward apparent enrichment for any pathway given the platform’s own non-random gene selection). Twenty-two Gene Ontology (GO) Biological Process, GO Molecular Function, and Reactome terms reached FDR < 0.05 (Table 5, Figure 3). The strongest single term was RNA Binding (GO:0003723; 13/37 genes overlapping—CPSF7, XRCC6, FUS, SRSF1, HNRNPDL, PUF60, EWSR1, FUBP1, G3BP2, LONP1, ZNF207, ERH, HNRNPA1; odds ratio [OR] = 6.43, FDR = 1.6 × 10^−4^), consistent with the descriptive “RNA-binding/nuclear protein” characterization. Reactome mRNA splicing pathways were independently enriched (mRNA Splicing, OR = 14.3, FDR = 0.0063; Processing of Capped Intron-Containing Pre-mRNA, OR = 11.5, FDR = 0.011), driven by the FET-family and heterogeneous nuclear ribonucleoprotein (hnRNP)/serine-arginine-rich splicing factor (SRSF) genes noted above. Two additional, previously undescribed categories also reached significance: nuclear envelope/lamina biology (Depolymerisation of Nuclear Lamina, Initiation of Nuclear Envelope Reformation, Protein Localization to Nuclear Envelope, Nuclear Envelope Organization; driven by LMNB2, TMPO, lamin A/C [LMNA]) and mitotic spindle/cell-cycle checkpoint signaling (Mitotic Spindle Checkpoint Signaling, Spindle Assembly Checkpoint Signaling, Negative Regulation of Mitotic Metaphase/Anaphase Transition; driven by cell division cycle 20 [CDC20] and zinc finger protein 207 [ZNF207]). These data refine the prior descriptive characterization: the monitor signature reflects not only RNA-binding/splicing machinery but also nuclear-envelope breakdown and mitotic-checkpoint components. This pattern is compatible with release of intracellular contents during cell division, mitotic stress, or cell death, but enrichment analysis alone cannot determine whether such release originates from metastatic tumor cells, the metastatic-site microenvironment, or nonspecific systemic cell turnover.

### 2.6. Exploratory Elastic-Net Composite Prediction Panel: Modest Internal Performance, Near-Chance External Validation

**Figure 4:**
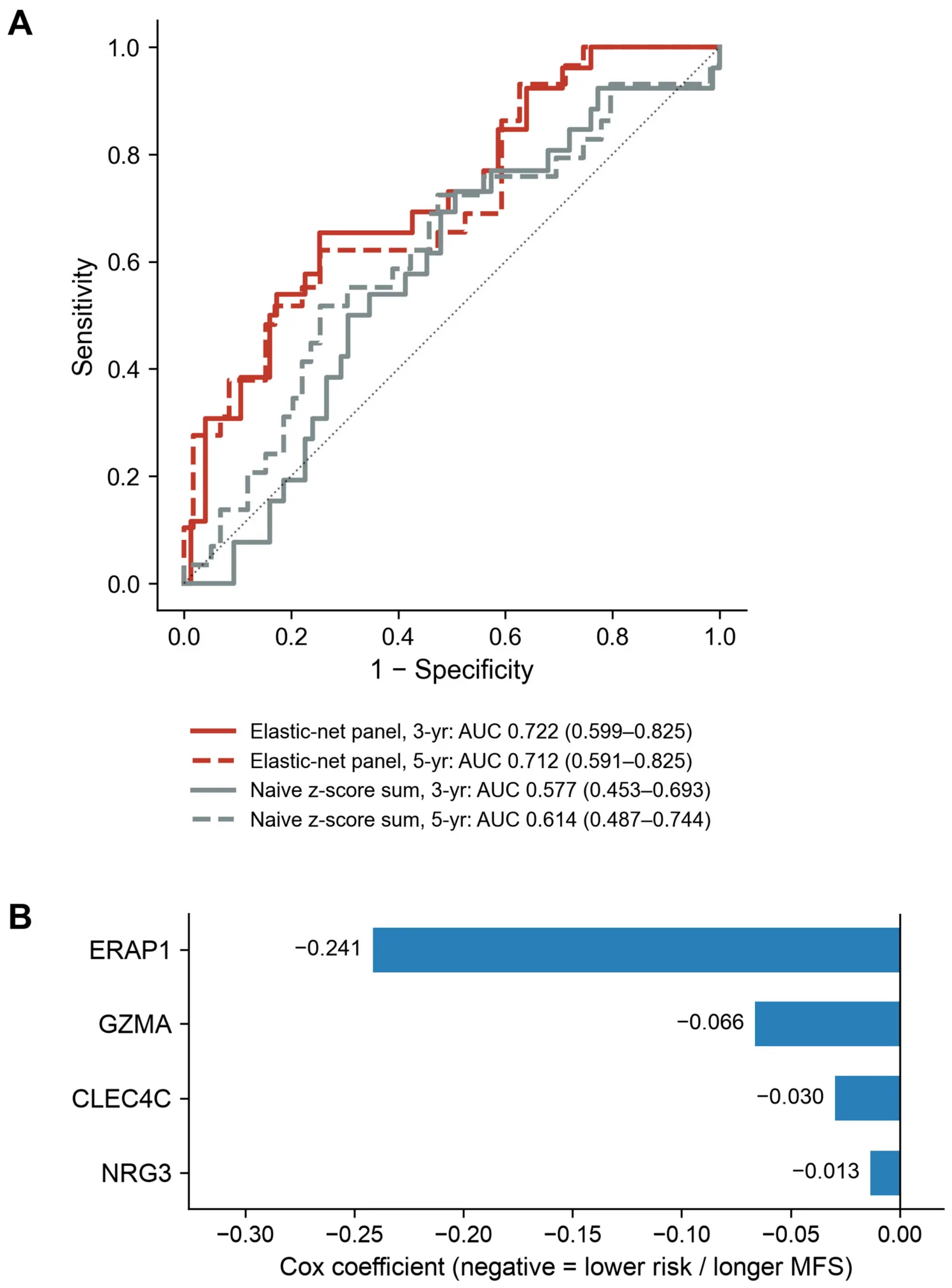
Elastic-net composite prediction-model panel (ERAP1, GZMA, CLEC4C, NRG3; l1 ratio = 0.5), fitted in the GSE103091 transcriptomic cohort (n = 107 with metastasis-free survival annotation, 31 events) using the serum-nominated candidate gene space. (**A**) Time-dependent receiver operating characteristic performance of the locked panel versus a naive z-score-sum composite in GSE103091 at 3 and 5 years. These are in-sample (apparent) AUCs because the locked model was scored on its own training cohort; nested cross-validation AUCs are reported in Table 6B; the nested five-fold cross-validation C-index within GSE103091 was 0.613. (**B**) Panel coefficients. External validation results are reported in Table 6; the panel is not yet a validated portable predictor beyond GSE103091.

Because the serum cohort has no time-to-event endpoint, we fitted an exploratory composite panel in a public tumor-tissue transcriptomic cohort, using as the candidate space the 24 prediction-model genes nominated by the serum limma analysis (23 with a matching GPL570 probe; Section 4.8). In GSE103091 [10] (n = 107 TNBC samples with metastasis-free survival annotation, 31 events), an elastic-net model (l1 ratio = 0.5) retained four genes (ERAP1, GZMA, CLEC4C, and NRG3) and achieved a nested cross-validation C-index of 0.613, comparable to a LASSO alternative (C-index = 0.637) and outperforming a naive unweighted z-score sum of the same four genes (C-index = 0.604). The small difference between the two penalized approaches is not statistically distinguishable at this sample size (n = 107, 31 events) and should not be over-interpreted; both substantially outperform the unweighted composite, motivating the use of a regularized rather than naive scoring approach. We report the elastic-net panel, as it directly generalizes the naive composite (l1 ratio = 0.5 vs. 1.0) and permits a moderately larger retained gene set at the locked, interpretable penalty. CLEC4C and NRG3 were model-selected features rather than top univariate serum DEP hits. External validation in GSE21653 [11] (n = 74, 21 events) yielded a C-index of 0.559—only marginally above the chance value of 0.5 and substantially below the internal nested-CV estimate. These data suggest the four-gene composite, as currently specified, is not yet a portable predictor and should not be presented or interpreted as a validated clinical panel. Panel performance and coefficients are shown in Figure 4, with full composition, cross-validation, and external-validation results in Table 6.

### 2.7. Supportive Tissue-Transcriptomic Analyses: GZMA mRNA Is Associated with Recurrence in Two Independent Cohorts; Monitor Hits Show Near-Chance Tissue Concordance

**Figure 5:**
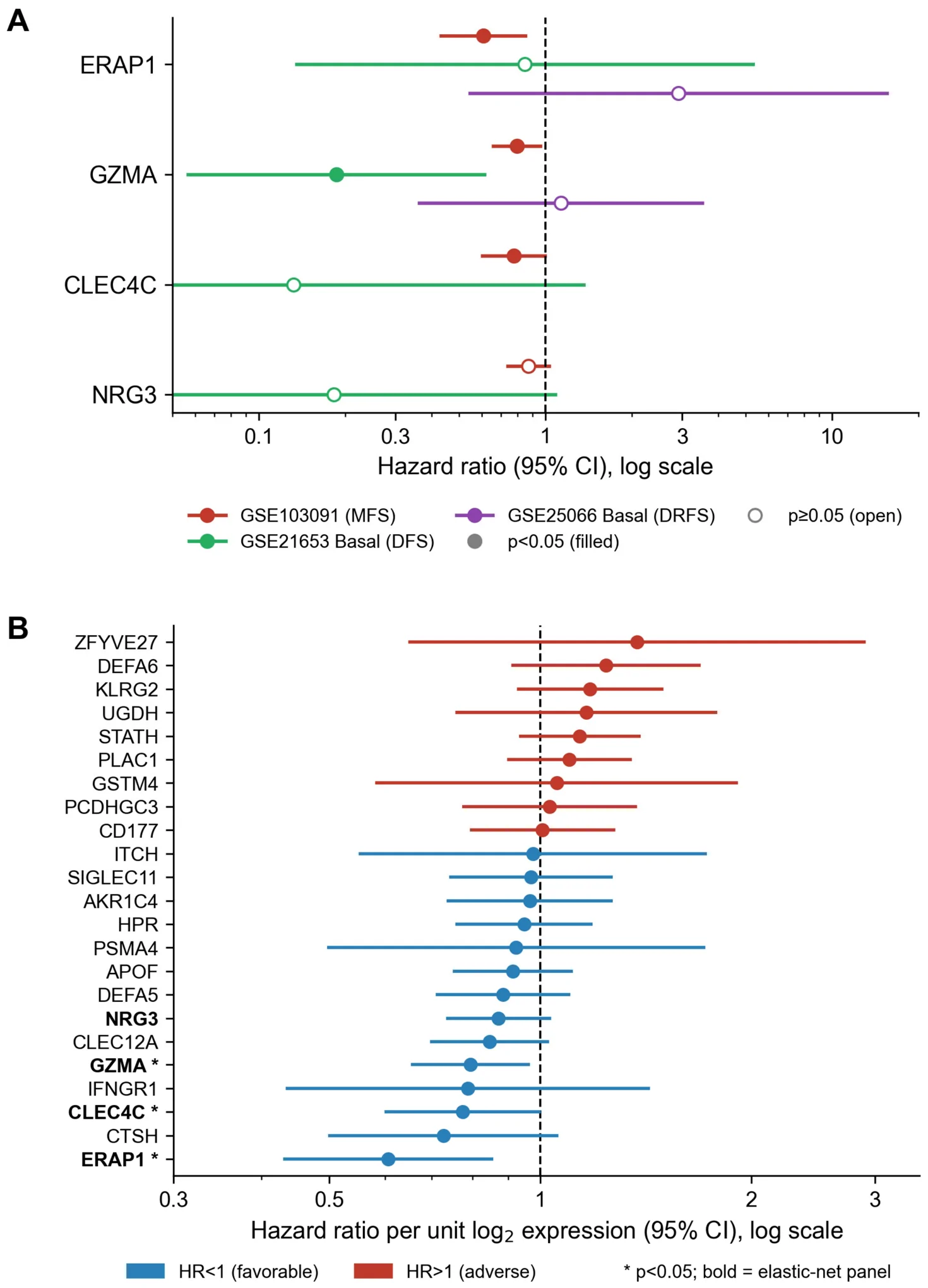
Cox proportional-hazards forest plots for prediction-model candidate genes in independent GEO transcriptomic cohorts with survival annotation. (**A**) HR with 95% CI for the four elastic-net panel genes (GZMA, ERAP1, CLEC4C, and NRG3) across GSE103091 (n = 107, metastasis-free survival [MFS]), GSE21653 (n = 74, Basal-subtype, disease-free survival [DFS]), and GSE25066 (n = 189, Basal-subtype, distant recurrence-free survival [DRFS]). (**B**) All 23 candidate genes tested in GSE103091. GZMA was significant in GSE103091 and GSE21653; ERAP1 and CLEC4C were significant in GSE103091 only; NRG3 showed a borderline association in GSE21653 (*p* = 0.061). * *p* < 0.05.

As a supportive analysis, we tested whether tumor-tissue mRNA expression of the prediction-model candidates was associated with breast cancer outcome in three independent GEO transcriptomic cohorts with survival annotation: GSE103091 [10] (n = 107 metastasis-free survival [MFS]-annotated TNBC samples), GSE21653 [11] (n = 74 Basal-subtype disease-free survival [DFS]-annotated samples), and GSE25066 [12] (n = 189 Basal distant recurrence-free survival [DRFS]-annotated samples). Briefly, because distant recurrence is, with rare exception, preceded by occult metastatic spread, we selected three independent GEO cohorts specifically for their metastasis- or recurrence-related survival annotation—metastasis-free survival (GSE103091), distant recurrence-free survival (GSE25066), and disease-free survival (GSE21653)—rather than for sample size or platform alone. Higher GZMA expression was significantly associated with longer recurrence-free survival in two independent cohorts: GSE103091 (HR = 0.795, 95% confidence interval [CI] 0.657–0.961, *p* = 0.018) and GSE21653 (HR = 0.186, 95% CI 0.057–0.614, *p* = 0.0057) (Figure 5A; Table 7). ERAP1 was significant in GSE103091 (HR = 0.606, 95% CI 0.432–0.852, *p* = 0.0039) but not in GSE21653 (*p* = 0.86) or GSE25066 (*p* = 0.21), indicating a cohort-specific association. Among the other elastic-net panel members, CLEC4C was significant in GSE103091 (HR = 0.775, *p* = 0.046), and NRG3 showed a borderline association (0.05 ≤ *p* < 0.10) in GSE21653 (HR = 0.183, *p* = 0.061); neither was significant in any other cohort. GZMA is therefore the candidate with the most consistent tissue-level support in this dataset, with concordant direction in two of three cohorts and 95% CIs excluding 1 in both. The complete candidate-gene forest plot for GSE103091 (all 23 genes tested; note that PDIA5 was removed because there was no matching probe on the GPL570 array) is shown in Figure 5B. The 23 genes carried into this validation step were drawn from an earlier, limma-based nominal list for the same prediction comparison. Of the four elastic-net panel members (Section 2.6), GZMA, ERAP1, and CLEC4C were significant in GSE103091, and NRG3 showed a borderline association in GSE21653. We note that CLEC4C’s direction is internally discordant: it was serum-elevated in the diagnosis-timepoint samples of patients who later recurred (the higher-risk DR-DIG group), yet higher tissue expression in GSE103091 was associated with more favorable, not worse, survival (HR < 1). We confirmed this discordance is not a labeling or computational artifact by independently re-deriving both the serum differential-expression call and the tissue Cox model, including both annotated CLEC4C probes on GPL570, which agree in direction.

**Figure 6:**
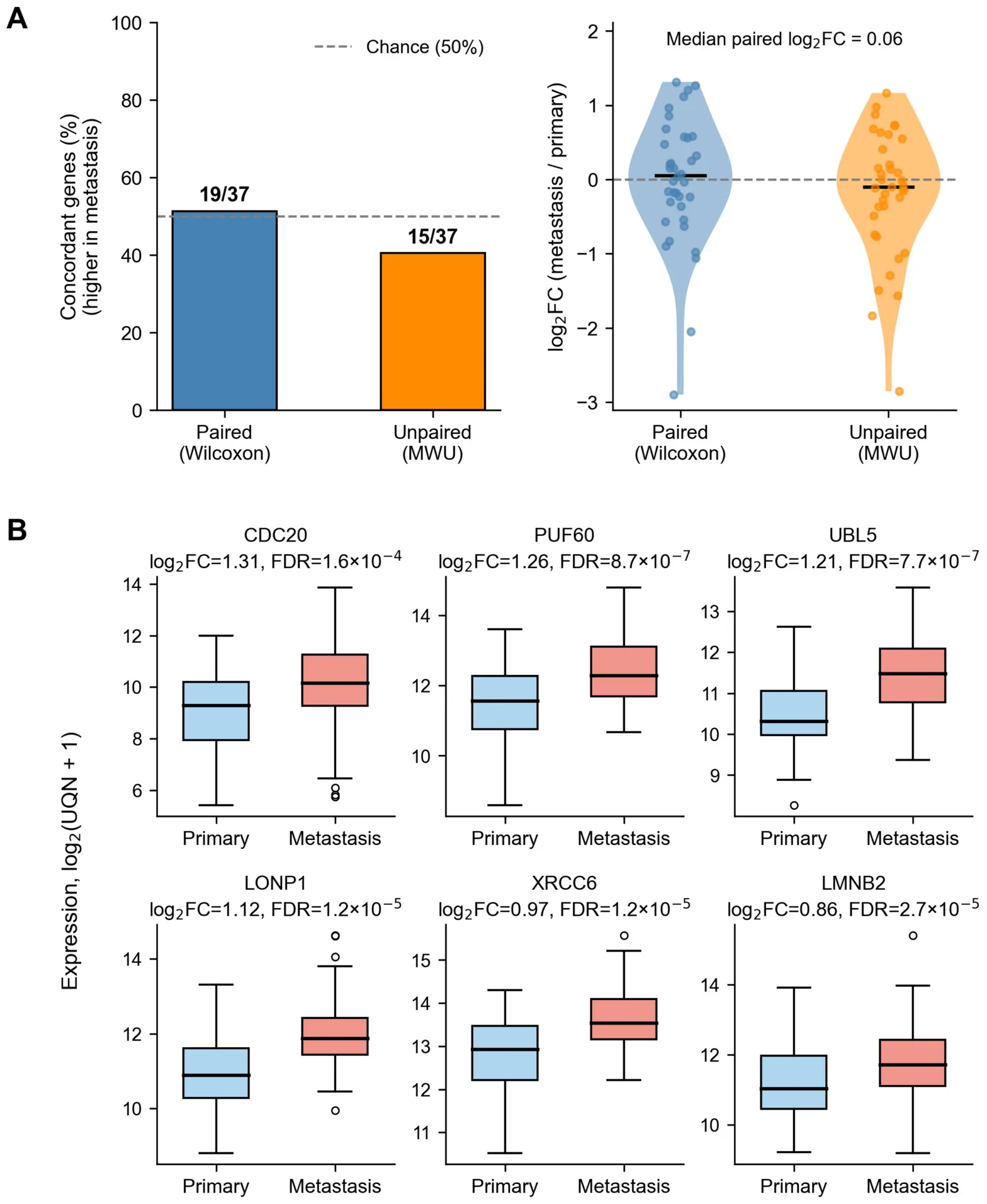
Monitor-model serum-vs-tissue concordance in GSE209998 (RNA-seq, 44 primary and 79 metastatic breast cancer tissue samples, 39 paired patients; no TNBC-specific subtype annotation). (**A**) Paired fold-change concordance for all 37 unique monitor-hit genes (19/37 concordant, 51.4%—near chance). (**B**) The six monitor genes with the largest concordant paired fold-change, each significantly upregulated in metastatic tissue (paired Wilcoxon signed-rank test, Benjamini–Hochberg false discovery rate < 0.05); 11 of the 37 genes met this criterion in total.

For the monitor hits, we tested transcriptional concordance in GSE209998 (44 primary and 79 metastatic breast cancer tissue samples, 39 matched pairs; no TNBC-specific subtype annotation available). Of 37 monitor genes tested (matched by symbol), 19 (51%) showed concordant direction (paired fold-change > 0, i.e., transcriptionally higher in metastatic tissue, matching the serum higher-in-DR-REC direction, Figure 6A), near chance level. This result argues against uniform transcriptional induction of the signature in metastatic tissue, but it does not distinguish release from metastatic deposits from nonspecific systemic cell turnover. Eleven of the 37 genes did show significant, concordant transcriptional elevation in metastatic tissue (paired Wilcoxon signed-rank test, Benjamini–Hochberg FDR < 0.05): UBL5 (paired log_2_FC = +1.21, FDR = 7.7 × 10^−7^), PUF60 (+ 1.26, FDR = 8.7 × 10^−7^), XRCC6 (+ 0.97, FDR = 1.2 × 10^−5^), LONP1 (+ 1.12, FDR = 1.2 × 10^−5^), LMNB2 (+ 0.86, FDR = 2.7 × 10^−5^), CDC20 (+ 1.31, FDR = 1.6 × 10^−4^), ABHD14A (+ 0.58, FDR = 2.7 × 10^−4^), LMNA (+ 0.68, FDR = 3.2 × 10^−4^), ERH (+ 0.56, FDR = 2.8 × 10^−3^), DPY30 (+ 0.47, FDR = 1.3 × 10^−2^), and EFNA3 (+ 0.58, FDR = 1.8 × 10^−2^); the six with the largest paired fold-changes are shown in Figure 6B. These eleven genes are candidates for a tumor- or metastasis-associated contribution to the serum signal and are prioritized for follow-up validation. A comparable number of monitor genes (nine of 37) were significantly discordant, that is, transcriptionally lower in metastatic tissue, so the FDR-significant genes divide near-evenly by direction, consistent with the near-chance overall concordance rate. In contrast, FUS was discordant between compartments (serum higher in DR-REC; metastatic tissue log_2_FC = −1.06, *p* < 0.001); for this gene, the serum increase is not explained by higher transcript levels in metastatic tissue.

## 3. Discussion

These data identify GZMA as a candidate serum protein biomarker of TNBC distant-recurrence risk, supported by concordant transcript-level associations with recurrence in two of three independent tumor-tissue cohorts, with 95% confidence intervals excluding the null in both. Because the GEO analyses test tumor-tissue mRNA rather than serum protein, this support is indirect. ERAP1 showed a comparably strong signal at diagnosis in the discovery serum cohort but was associated with outcome in only one of three independent tissue cohorts, illustrating—within a single study—the distinction between a discovery-cohort hit and a portable biomarker candidate. Both genes point toward an immune-surveillance mechanism: GZMA is a cytotoxic T-lymphocyte and natural killer-cell effector protease, and ERAP1 trims peptides for MHC-I antigen presentation. These data suggest, but do not establish, that a more robust anti-tumor immune surveillance state at diagnosis distinguishes patients whose TNBC will not go on to recur; we make no causal claim, as the current design (case-control at a single timepoint, single biofluid) cannot distinguish cause from correlate.

For the monitor comparison, these data suggest a 38-aptamer serum signature, retained after exclusion of de novo Stage IV samples, accompanies clinically apparent distant recurrence in previously diagnosed TNBC. Exploratory over-representation analysis supported the descriptive characterization of this signature as RNA-binding/spliceosomal and additionally identified nuclear-envelope and mitotic-checkpoint enrichment. Combined with (1) the finding that most of these 38 proteins are not classically annotated as secreted, (2) near-chance (51%) transcriptional concordance with independent metastatic-tissue transcriptomic data, and (3) at least one gene (FUS) with directly discordant serum-versus-tissue direction, these data suggest that much of the monitor signature reflects release of intracellular protein content rather than a coordinated secretory program. The source of that release cannot be determined from the present data. Several explanations remain possible: release from metastatic deposits or the metastatic-site microenvironment, nonspecific systemic cell turnover, treatment-related tissue injury, and pre-analytical leakage during sample handling. We did not assay cell death or turnover directly, and we regard cell turnover or lysis as one candidate explanation rather than the favored mechanism.

Eleven monitor genes (UBL5, PUF60, XRCC6, LONP1, LMNB2, CDC20, ABHD14A, LMNA, ERH, DPY30, EFNA3) showed both higher serum abundance in the DR-REC group and independently significant, concordant transcriptional elevation in metastatic tissue, distinguishing them from the majority of the panel and identifying them as the more plausible candidates for a tumor- or metastasis-associated contribution. To pursue this directly, we tested whether primary-tumor expression of these eleven genes is itself prognostic, applying the same Cox proportional-hazards approach used for the prediction-model candidates to the same three GEO survival cohorts. Ten of eleven genes were analyzable in GSE103091 (ABHD14A had no matching GPL570 probe). Two reached nominal significance, both in the adverse direction expected of a tumor-derived marker—higher primary-tumor expression associated with shorter metastasis-free survival: LONP1 (HR = 2.598, 95% CI 1.438–4.696, *p* = 0.0016) and EFNA3 (HR = 1.563, 95% CI 1.090–2.241, *p* = 0.0152); LMNA was borderline (HR = 1.546, 95% CI 0.974–2.453, *p* = 0.0647). Neither LONP1 nor EFNA3 reached significance in the two independent replication cohorts (GSE21653, GSE25066), but the confidence intervals there were extremely wide (e.g., LONP1 in GSE21653: HR = 4.378, 95% CI 0.062–309.544, *p* = 0.497), consistent with insufficient events to resolve an effect of this size rather than clear evidence against one. This single-cohort, non-replicated result carries the same epistemic status as ERAP1′s prediction-model finding above: it is weak, hypothesis-generating evidence directionally consistent with a genuine tumor contribution for at least a subset of the monitor signature and requires confirmation in a cohort with more distant-recurrence events before it can support a stronger claim.

Two further components of the signature are compatible with a tumor- or microenvironment-associated contribution. The mitotic-checkpoint proteins CDC20 and ZNF207 are consistent with, but not specific to, a proliferating tumor-cell source. CXCL14, one of the four monitor hits that passed the secretome/cancer blood-proteome filter, is a secreted chemokine expressed by breast cancer epithelial cells and by cancer-associated fibroblasts [13,14], although its role in TNBC appears context-dependent [15]. Its elevation at recurrence is therefore consistent with, but not specific to, a tumor-microenvironment source. None of these observations exclude the alternative explanations above. The tissue data are bulk transcriptomic data from a mixed-subtype cohort, and the serum samples are not paired with tissue from the same patients. Resolving the source of the monitor signature will require paired serum and metastatic-tissue biopsy sampling from the same TNBC patients at recurrence.

A design feature of the monitor comparison warrants explicit discussion rather than implicit assumption: DR-DIG and DR-REC are predominantly different patients, not paired longitudinal samples from the same individuals—only one patient contributed both a baseline and a recurrence-timepoint sample. This is a between-group, not within-patient, comparison, and the biological interpretation above should be read accordingly: the 38-protein signature reflects a difference between two largely distinct groups of TNBC patients at two different disease states, which is a weaker inferential position than a true within-patient before/after comparison would provide. We directly tested whether this design could instead reflect an assay-batch or plate/run-date artifact rather than biology: all 40 cohort samples, including every DR-DIG and DR-REC sample, were processed on a single plate on a single run date, and neither physical slide-position distribution nor a per-sample hybridization-normalization QC metric differed between groups. This assay-batch confound is therefore excluded. We also examined pre-analytical and clinical factors using records obtained from the BORN-Biobank (Supplementary Table S2). All samples were collected before treatment, and no patient in any group had an active infection or hepatic or renal dysfunction at the time of collection. Serum storage duration did not differ significantly between the DR-DIG and DR-REC groups (median 5.0 vs. 10.0 years; Mann–Whitney U *p* = 0.42), and the signature score did not correlate with storage duration among the 20 samples from patients without recurrent disease (Spearman ρ = 0.18, *p* = 0.46). All 38 hits remained higher in DR-REC after adjustment for storage duration (*p* < 0.05 for all; median attenuation of the group effect 9%, maximum 14%), although nine of the 38 aptamers showed a nominal association with storage duration, so a storage effect cannot be fully excluded (Supplementary Table S4). All 38 hits also remained higher in DR-REC when each DR-REC sample, including one from a patient with myelodysplastic syndrome, was left out in turn (Supplementary Table S3). Concomitant medications differed between groups; notably, analgesics (non-steroidal anti-inflammatory drugs or opioids) were recorded only in DR-REC. Other pre-analytical factors could not be evaluated: the time from collection to processing and freezing was not documented for older samples, and freeze–thaw history and hemolysis were not recorded. Because the monitor signature’s protein composition (RNA-binding, nuclear-envelope, and mitotic-checkpoint proteins; above) is plausibly sensitive to sample handling as well as to cell-turnover biology, these data reduce, but do not eliminate, this concern, which we address in the limitations below.

The elastic-net four-gene composite panel (ERAP1, GZMA, CLEC4C, NRG3) illustrates a caution we consider important to state plainly rather than minimize: its promising internal, nested-cross-validation C-index (0.613) did not hold up in external validation (0.559, only marginally above chance). A LASSO alternative fit on the same candidate space achieved a nominally higher nested-cross-validation C-index (0.637); we do not read this as evidence that LASSO is the superior approach, since the two estimates were not formally compared against one another (e.g., via a paired test across the five outer cross-validation folds) and, at this sample size (n = 107 with 31 events), a difference of this magnitude is well within what cross-validation fold assignment alone could produce. Both penalized approaches substantially outperformed the naive unweighted composite (C-index = 0.604)—the more robust conclusion this comparison supports is that regularization improves on an unweighted sum, not that either specific penalty is superior to the other. Because the candidate space was nominated in the serum discovery cohort and the panel members were then selected and fitted within the same transcriptomic cohort (GSE103091) that supplied the internal cross-validation estimate, some of the internal–external gap reflects expected selection optimism rather than a failure specific to this panel; nonetheless, we do not consider the composite panel, in its current form under either penalty, ready for prospective evaluation, and we explicitly do not claim it has clinical validity. GZMA alone—the one panel member with transcript-level support in two independent cohorts—is the more defensible candidate to carry forward. A further caution specific to CLEC4C: its serum-discovery direction (elevated in the higher-risk DR-DIG group) and its tissue hazard ratio (protective, HR < 1 in GSE103091) point in opposite directions. This discordance is not unique to CLEC4C—roughly half of the 24 nominal prediction candidates show similarly discordant serum-versus-tissue directions in GSE103091, consistent with most of this nominal (non-FDR-significant) list reflecting statistical noise rather than reproducible biology—but it is particularly relevant here because CLEC4C is one of only four genes retained in the locked composite panel. It may also reflect genuine biological decoupling: CLEC4C (CD303/BDCA-2) marks plasmacytoid dendritic cells rather than tumor cells, so systemic serum levels and tumor-infiltrating tissue levels of this marker are not obligated to move together. We flag this as a further reason to treat the composite panel’s individual members, not only its aggregate external-validation performance, with caution.

We identify at least five concrete limitations of this study, each paired with the specific follow-up experiment that would address it. First, and most consequential for the monitor-model finding specifically, the monitor comparison is between largely different patients, and pre-analytical records were incomplete. Storage duration was known for all samples and did not explain the monitor signature, but the time from blood collection to processing and freezing was not documented for older samples, freeze–thaw history and hemolysis status were not recorded, and storage duration ranged up to 13.6 years. Clinical factors such as concomitant medications also differed between groups, so residual confounding cannot be excluded. This would be addressed by a prospective cohort in which serum is collected under a standardized protocol, with clinical and pre-analytical variables recorded at each draw, ideally with paired baseline and recurrence samples from the same patients (this cohort provided only one such pair). Second, the discovery cohort is small (8–12 samples per group), and the prediction comparison in particular is underpowered—zero proteins reached FDR significance despite a biologically coherent nominal signal (GZMA, ERAP1). This would be directly addressed by profiling a larger, prospectively collected TNBC diagnosis-timepoint serum cohort with sufficient recurrence events to reach FDR significance for a candidate panel restricted a priori to GZMA and ERAP1. Third, the elastic-net composite panel’s gene selection and its cross-validation estimate both derive from the same discovery cohort, producing optimism that is only partly correctable by nested cross-validation; this would be directly addressed by locking the panel’s gene composition and coefficients before testing in a second, fully independent, prospectively collected cohort—precisely the external-validation step already attempted here (GSE21653) and already found wanting, underscoring rather than merely predicting the need for a properly powered second cohort. Fourth, the monitor-model’s supportive tissue analysis compares serum protein abundance to bulk tissue transcript abundance in a cohort (GSE209998) with no TNBC-specific subtype annotation, diluting any TNBC-specific signal within a mixed-subtype breast cancer population; this would be directly addressed by validating the monitor signature in a subtype-annotated primary-versus-metastatic transcriptomic cohort or—more directly—by pairing serum and metastatic-tissue biopsy sampling within the same TNBC patients at the time of recurrence. Fifth, all candidates were identified in a single serum cohort, and the GEO analyses test tumor-tissue mRNA rather than serum protein; protein-level validation of GZMA, ERAP1, and the monitor signature in an independent serum cohort has not yet been performed. This would be addressed by measuring these candidates, with the analysis plan specified in advance, in an independent, prospectively collected TNBC serum cohort, which is the essential next step.

## 4. Materials and Methods

### 4.1. Study Cohort and Sample Groups

Serum samples were obtained from the BORN-Biobank (Kyoto University collaborators) TNBC cohort. Four groups were analyzed: healthy controls (n = 10), non-recurrent TNBC patients (NR, n = 12), diagnosis-timepoint serum from patients later found to develop distant recurrence (DR-DIG, n = 8), and recurrence-timepoint serum from patients with clinically apparent recurrence (DR-REC, n = 9). The DR-DIG and DR-REC groups are largely composed of different patients: cross-referencing the analyzed sample groupings against patient identifiers confirmed that only one patient contributed both a diagnosis-timepoint and a recurrence-timepoint sample; a second candidate pair was not realized because the sample collected at diagnosis was excluded for quality-control failure, leaving the sample collected at recurrence unpaired in DR-REC. The monitor comparison is therefore a between-group (predominantly between-patient) comparison rather than a within-patient longitudinal design; this is addressed directly in Discussion and Limitations. Four DR-REC samples represented de novo AJCC Stage IV disease (metastatic at presentation); three of these carried a diagnosis-timepoint designation in the biobank annotation and were reclassified into DR-REC, and the fourth was already designated a recurrence-timepoint sample. A sensitivity analysis excluding these four samples (true distant-recurrence-only comparison, n = 5 vs. 8) was performed for the monitor model. All n’s reported are biological replicates (one serum sample per patient per timepoint); no technical replicate pooling was performed. All TNBC serum samples were collected before treatment: NR and DR-DIG samples at diagnosis, before any treatment; the five distant-recurrence DR-REC samples at the diagnosis of recurrence, before treatment for recurrent disease; and the four de novo Stage IV samples at initial diagnosis, before systemic therapy. For every analyzed TNBC sample, the BORN-Biobank investigators reviewed the medical records for comorbidities, concomitant medications, active infection, and hepatic or renal dysfunction at the time of collection and provided the collection and biobank release dates, from which storage duration was calculated (Supplementary Table S2). All samples were released from the biobank on the same date. Sex as a biological variable is not applicable because the BORN-Biobank TNBC cohort contains no male breast cancer.

### 4.2. Pre-Analytical/Assay-Batch Confound Check (Monitor Model)

Since DR-DIG and DR-REC are predominantly different patients, we tested whether assay batch or plate/run-date could confound the monitor comparison—i.e., whether the two groups were processed differently rather than differing biologically. All 40 samples in the SomaScan delivery (the 39 analyzed samples plus the one quality-control failure), including every DR-DIG and DR-REC sample, were assayed on a single SomaScan plate on a single run date; there is no second batch or run date in this delivery, so an assay-batch/plate confound is directly excluded by construction. Physical slide-position distribution of DR-DIG vs. DR-REC samples across the plate showed no evidence of group-level clustering (chi-square test, exploratory given the small expected cell counts: χ^2^ = 6.30, df = 6, *p* = 0.391), and the per-sample hybridization-normalization QC metric (HybControlNormScale) did not differ between groups (Mann–Whitney U, DR-DIG median 1.041 vs. DR-REC median 1.022, *p* = 0.541); all 39 analyzed samples passed the platform RowCheck QC flag. The robustness of the 38 monitor hits to individual samples and to storage duration was assessed by (i) leave-one-out analysis over each DR-REC sample (two-sided Mann–Whitney U test per aptamer); (ii) linear regression of log_2_ abundance on group (DR-REC vs. DR-DIG) with storage duration (years) as a covariate; and (iii) comparison of DR-DIG with the DR-REC samples whose storage duration fell within the DR-DIG range. The association between storage duration and a per-sample signature score (mean z-score of the 38 aptamers relative to DR-DIG) was tested by Spearman correlation. These analyses were exploratory.

### 4.3. SomaScan Proteomic Profiling and Normalization

Serum protein abundance was measured using the SomaScan v5.0 aptamer-based platform (11,083 aptamers, of which 10,788 target human proteins). Relative fluorescence unit values were ANML-normalized per manufacturer protocol and log_2_-transformed prior to analysis. Analysis was restricted to the 10,551 human aptamers passing platform-level quality control (ColCheck = PASS), corresponding to 9482 unique gene symbols.

### 4.4. Differential Expression Protein (DEP) Analysis

Two pairwise comparisons were defined: prediction (NR, n = 12, vs. DR-DIG, n = 8) and monitor (DR-DIG, n = 8, vs. DR-REC, n = 9). Three statistical tests were applied to each comparison for method comparison and sensitivity assessment: Mann–Whitney U (two-sided, unpaired), Welch’s *t*-test, and a moderated *t*-test (limma, empirical Bayes variance shrinkage, ‘trend = TRUE’)—the last designated as the PRIMARY test following observation that Mann–Whitney U produces discrete rank-tie artifacts and Welch’s *t*-test yields unstable per-gene variance estimates at this sample size (n = 8–9 per group). Benjamini–Hochberg FDR correction was applied within each test. FDR < 0.05 defined a significant hit. For the prediction comparison, a nominal discovery threshold (limma *p* < 0.05 AND |log_2_FC| ≥ 0.5) was additionally applied given the absence of FDR-significant hits, yielding 25 aptamers (24 unique genes). A sensitivity analysis for the monitor model excluded the four de novo Stage IV samples described above and re-tested nominal significance and rank separation for all 38 primary hits.

### 4.5. Secretome Filtering

The 38 Limma-FDR Monitor Hits and the 25 Limma-Nominal Prediction hits were filtered against two primary reference sources (HPA plasma secretome [7] and HPA cancer proteome [8]). The HPA plasma secretome Data File S2, “The human secretome genes and their annotated categories,” is the systematic secretome classification (2640 genes; annotated categories include Secreted to blood [n = 730], Intracellular or membrane-bound [n = 932], four locally secreted sub-categories [n = 643 combined], and Secreted-unknown location [n = 173]). The HPA cancer proteome BioStudies deposit (accession S-BSST935) is a pan-cancer blood-proteome PROXIMITY EXTENSION ASSAY (PEA) measurement dataset (1463 proteins, 12 cancer types including breast cancer, ~2.08 million measurements), not a secretome classification—its own supplementary data files contain no secreted-protein annotation table. This filter accordingly defines: secreted = HPA plasma secretome; plasma-proteome-detected = gene present among the 1463 proteins measured in HPA cancer proteome panel. Housekeeping exclusion and hematopoietic/blood-lineage exclusion [9] (HPA “RNA blood lineage specificity” = “Lineage enriched,” “Lineage enhanced,” or “Group enriched”) were also applied; because neither HPA plasma secretome nor HPA cancer proteome provides a blood-lineage-specificity field, the general HPA bulk annotation download was used for that one exclusion criterion only.

### 4.6. Pathway/Over-Representation Analysis

Over-representation analysis was performed on the 37 unique gene symbols comprising the 38 limma-FDR monitor hits using gseapy’s Enrichr interface (Fisher’s exact test, Benjamini–Hochberg FDR) against GO-Biological-Process-2023, GO-Molecular-Function-2023, and Reactome-2022 gene-set libraries. The background gene set was restricted to the 9482 unique gene symbols measured across all 10,551 SomaScan v5.0 aptamers (rather than Enrichr’s default whole-genome background) to avoid bias from the platform’s own non-random gene selection. FDR < 0.05 defined a significant term.

### 4.7. Supportive Tissue-Transcriptomic Analyses (GEO)

Prediction-model candidates were tested for Cox proportional-hazards association with survival in three independent public GEO cohorts: GSE103091 (n = 107 TNBC, MFS; combined GSE58812 + GSE83937 series), GSE21653 (n = 74 Basal-subtype, DFS), and GSE25066 (n = 189 Basal-subtype, DRFS). Cox models report HR, 95% CI, and *p*-value per gene per cohort. Cohort endpoint selection for these supportive analyses was guided by the clinical relationship between distant recurrence and metastatic disease: with the exception of patients presenting with de novo metastatic (Stage IV) disease at diagnosis, distant recurrence is preceded by occult metastatic spread, whereas not every disease recurrence progresses to distant metastasis. We therefore prioritized public cohorts with survival annotation specific to metastasis or distant recurrence over the broader disease-free survival endpoint. GSE103091, annotated for MFS, served as the primary cohort. GSE25066, annotated for DRFS, provided an independent cohort with a directly matched endpoint. GSE21653, for which only DFS (encompassing locoregional as well as distant events) was available, was retained as a third independent replication cohort under the same rationale, since distant recurrence is itself a subset of disease recurrence. Monitor-model candidates were tested for paired transcriptional concordance in GSE209998 (44 primary and 79 metastatic breast cancer tissue samples, 39 matched pairs, RNA-seq, upper-quartile-normalized [UQN], log_2_(UQN + 1)-transformed; no TNBC-specific subtype annotation available) using paired Wilcoxon signed-rank testing (mean-of-metastases-per-patient vs. primary) with Benjamini–Hochberg FDR correction and unpaired MWU as a secondary check [16].

### 4.8. Elastic-Net Composite Panel

An elastic-net penalized Cox model (l1 ratio = 0.5, penalty selected by nested cross-validation) was fitted in the GSE103091 transcriptomic cohort (n = 107 samples with metastasis-free survival annotation, 31 events) using as its candidate feature space the 24 prediction-model genes nominated by the serum limma analysis (23 with a matching GPL570 probe). The serum discovery cohort carries no time-to-event endpoint and was therefore not used to fit or to cross-validate the model; it contributed the candidate gene list only. Model performance was assessed by nested five-fold cross-validation C-index within GSE103091 compared against a LASSO alternative (l1 ratio = 1.0) and a naive unweighted z-score-sum composite of the same four genes. The locked four-gene panel (ERAP1, GZMA, CLEC4C, NRG3) was externally validated in GSE21653 (n = 74, 21 events; DFS endpoint).

### 4.9. Statistical Standards

All continuous two-group comparisons and their associated FDR values are reported together with the test used, the sample size, the effect size, the *p*-value, and the Benjamini–Hochberg FDR (Mann–Whitney U as the default two-group test; the limma moderated *t*-test as the explicitly justified primary test given small-n variance instability). Survival analyses report hazard ratio with 95% CI. Borderline *p*-values (0.05 ≤ *p* < 0.10) are flagged explicitly as borderline and are not interpreted as clean positive findings. All sample sizes reported are biological replicates.

## 5. Conclusions

In conclusion, these data nominate GZMA as a candidate serum biomarker for TNBC recurrence-risk stratification, supported by concordant transcript-level associations in two independent tissue cohorts; identify a preliminary monitor-model recurrence signature whose cellular source remains to be determined; and explicitly do not support clinical application of the elastic-net composite panel without further, independent, prospective validation. All candidates require protein-level validation in an independent serum cohort.

## Figures and Tables

**Table 1:** Cohort demographics and QC summary for the BORN-Biobank serum proteomic cohort. Treatment history is shown as yes/no/not reported (n).

Characteristic	Healthy Control	NR	DR-DIG	DR-REC
n	10	12	8	9
Median age, y (range)	60 (37–74)	63 (32–75)	61 (26–85)	59 (35–70)
Race, Asian, n (%)	10 (100%)	12 (100%)	8 (100%)	9 (100%)
Sex, female, n (%)	10 (100%)	12 (100%)	8 (100%)	9 (100%)
Stage at diagnosis (AJCC), Stage IA, n	N/A	2	0	0
Stage at diagnosis (AJCC), Stage IIA, n	N/A	3	1	2
Stage at diagnosis (AJCC), Stage IIB, n	N/A	2	2	0
Stage at diagnosis (AJCC), Stage IIIA, n	N/A	2	1	0
Stage at diagnosis (AJCC), Stage IIIB, n	N/A	1	0	1
Stage at diagnosis (AJCC), Stage IIIC, n	N/A	0	0	1
Stage at diagnosis (AJCC), Stage IV, n	N/A	0	0	4
Stage at diagnosis, not reported, n	N/A	2	4	1
Neoadjuvant chemotherapy, yes/no/not reported, n	N/A	4/8/0	3/5/0	2/5/2
Neoadjuvant immune checkpoint inhibitor, yes/no/not reported, n	N/A	0/9/3	0/7/1	0/4/5
Adjuvant therapy, yes/no/not reported, n	N/A	9/2/1	5/3/0	0/4/5
Adjuvant capecitabine, yes/no/not reported, n	N/A	2/9/1	2/5/1	0/2/7
Adjuvant immune checkpoint inhibitor, yes/no/not reported, n	N/A	1/10/1	0/8/0	0/2/7

**Table 2:** Full prediction-model nominal DEP hit list.

SeqId	Gene	log_2_FC	Direction	*p* (MWU)	FDR (MWU)	*p* (Welch)	FDR (Welch)	*p* (Limma)	FDR (Limma)
4964-67	ERAP1	−0.896	UP_NR	0.0005	1.0000	0.0006	0.9459	3.60 × 10^−5^	0.3793
29520-118	HPR	1.363	UP_DR-DIG	0.0041	1.0000	0.0008	0.9459	0.0010	0.9275
17755-5	UGDH	0.535	UP_DR-DIG	0.0011	1.0000	0.0226	0.9459	0.0027	0.9275
9094-5	CLEC4C	0.585	UP_DR-DIG	0.0096	1.0000	0.0027	0.9459	0.0035	0.9275
3440-7	GZMA	−0.592	UP_NR	0.0252	1.0000	0.0076	0.9459	0.0057	0.9275
8644-46	CTSH	0.643	UP_DR-DIG	0.0159	1.0000	0.0253	0.9459	0.0061	0.9275
6387-61	DEFA5	−0.764	UP_NR	0.0073	1.0000	0.0046	0.9459	0.0072	0.9275
16785-45	DEFA5	−0.656	UP_NR	0.0055	1.0000	0.0083	0.9459	0.0093	0.9275
13116-25	CD177	−0.750	UP_NR	0.0201	1.0000	0.0319	0.9459	0.0131	0.9275
18397-5	AKR1C4	0.696	UP_DR-DIG	0.0473	1.0000	0.0294	0.9459	0.0135	0.9275
29465-32	DEFA6	−0.713	UP_NR	0.0387	1.0000	0.0163	0.9459	0.0143	0.9275
9102-28	ZFYVE27	−0.544	UP_NR	0.1569	1.0000	0.0652	0.9459	0.0173	0.9275
32585-3	PLAC1	0.503	UP_DR-DIG	0.0314	1.0000	0.0717	0.9459	0.0188	0.9275
20538-71	SIGLEC11	−0.767	UP_NR	0.0387	1.0000	0.0492	0.9459	0.0214	0.9275
14099-20	PSMA4	0.588	UP_DR-DIG	0.0691	1.0000	0.0986	0.9459	0.0242	0.9275
20524-38	NRG3	−0.766	UP_NR	0.0201	1.0000	0.0181	0.9459	0.0246	0.9275
7859-21	PCDHGC3	0.600	UP_DR-DIG	0.0473	1.0000	0.0517	0.9459	0.0247	0.9275
17172-19	STATH	0.959	UP_DR-DIG	0.0473	1.0000	0.0834	0.9459	0.0264	0.9275
12551-3	ITCH	−1.304	UP_NR	0.0314	1.0000	0.0211	0.9459	0.0321	0.9275
5825-49	IFNGR1	−0.505	UP_NR	0.0073	1.0000	0.0219	0.9459	0.0334	0.9275
10693-43	KLRG2	−1.776	UP_NR	0.0124	1.0000	0.0268	0.9459	0.0403	0.9275
11187-11	CLEC12A	−0.645	UP_NR	0.0387	1.0000	0.0269	0.9459	0.0406	0.9275
12370-30	APOF	−0.680	UP_NR	0.0574	1.0000	0.0766	0.9459	0.0422	0.9275
5593-11	PDIA5	0.607	UP_DR-DIG	0.0691	1.0000	0.0574	0.9459	0.0478	0.9275
18895-54	GSTM4	−0.633	UP_NR	0.0979	1.0000	0.0367	0.9459	0.0491	0.9275

**Table 3:** Full monitor-model DEP hit list (n = 38 aptamers, 37 unique genes) reaching Benjamini–Hochberg FDR < 0.05 under the primary limma moderated *t*-test (empirical Bayes variance shrinkage), with MWU and Welch’s *t*-test *p*-values/FDR shown for method comparison.

SeqId	Gene	log_2_FC	Direction	*p* (MWU)	FDR (MWU)	*p* (Welch)	FDR (Welch)	*p* (Limma)	FDR (Limma)
13943-38	DPY30	2.402	UP_DR-REC	8.23 × 10^−5^	0.0299	9.64 × 10^−5^	0.1433	7.82 × 10^−6^	0.0361
20960-47	FUBP1	1.470	UP_DR-REC	8.23 × 10^−5^	0.0299	6.12 × 10^−5^	0.1433	1.07 × 10^−5^	0.0361
31440-109	DCDC2	1.286	UP_DR-REC	8.23 × 10^−5^	0.0299	8.06 × 10^−5^	0.1433	1.43 × 10^−5^	0.0361
6294-11	BAGE2	1.729	UP_DR-REC	0.0002	0.0354	4.97 × 10^−5^	0.1433	1.55 × 10^−5^	0.0361
10852-114	HNRNPDL	1.213	UP_DR-REC	8.23 × 10^−5^	0.0299	8.70 × 10^−5^	0.1433	1.72 × 10^−5^	0.0361
5715-4	ABHD14A	1.593	UP_DR-REC	0.0002	0.0354	0.0002	0.1433	2.83 × 10^−5^	0.0361
33622-23	ZNF394	1.347	UP_DR-REC	8.23 × 10^−5^	0.0299	0.0003	0.1433	2.84 × 10^−5^	0.0361
31035-50	SPIRE2	1.059	UP_DR-REC	8.23 × 10^−5^	0.0299	0.0004	0.1528	3.26 × 10^−5^	0.0361
15693-9	PGR	1.481	UP_DR-REC	8.23 × 10^−5^	0.0299	0.0002	0.1433	3.87 × 10^−5^	0.0361
25814-34	ZNF207	1.608	UP_DR-REC	8.23 × 10^−5^	0.0299	0.0003	0.1433	4.18 × 10^−5^	0.0361
26209-2	SRSF1	1.655	UP_DR-REC	0.0002	0.0354	0.0003	0.1433	4.40 × 10^−5^	0.0361
6256-9	LY6G6C	1.215	UP_DR-REC	0.0006	0.0528	0.0002	0.1433	4.43 × 10^−5^	0.0361
28494-11	CDC20	1.091	UP_DR-REC	0.0006	0.0528	0.0002	0.1433	4.45 × 10^−5^	0.0361
6398-12	LONP1	1.343	UP_DR-REC	0.0003	0.0408	0.0001	0.1433	4.78 × 10^−5^	0.0361
6296-36	OIT3	1.428	UP_DR-REC	0.0003	0.0408	0.0002	0.1433	5.67 × 10^−5^	0.0380
6291-55	CLSTN3	1.632	UP_DR-REC	8.23 × 10^−5^	0.0299	0.0003	0.1433	6.20 × 10^−5^	0.0380
5730-60	CXCL14	0.589	UP_DR-REC	8.23 × 10^−5^	0.0299	0.0004	0.1598	7.45 × 10^−5^	0.0380
10370-21	STAT1	0.774	UP_DR-REC	0.0002	0.0354	0.0002	0.1433	7.61 × 10^−5^	0.0380
6036-78	EPHA10	1.739	UP_DR-REC	0.0003	0.0408	0.0003	0.1433	7.76 × 10^−5^	0.0380
29942-8	CPSF7	0.923	UP_DR-REC	0.0002	0.0354	0.0002	0.1433	7.78 × 10^−5^	0.0380
12988-49	EWSR1	1.469	UP_DR-REC	8.23 × 10^−5^	0.0299	0.0005	0.1598	8.76 × 10^−5^	0.0380
29826-44	FUS	1.661	UP_DR-REC	0.0002	0.0354	0.0005	0.1598	8.84 × 10^−5^	0.0380
33227-149	FAM161A	1.493	UP_DR-REC	0.0003	0.0408	0.0004	0.1581	8.92 × 10^−5^	0.0380
10575-31	PUF60	1.716	UP_DR-REC	8.23 × 10^−5^	0.0299	0.0006	0.1598	9.01 × 10^−5^	0.0380
12466-7	HNRNPA1	0.952	UP_DR-REC	0.0003	0.0408	0.0003	0.1433	9.65 × 10^−5^	0.0381
6290-3	UTS2B	0.551	UP_DR-REC	0.0003	0.0408	0.0005	0.1598	0.0001	0.0381
21104-37	UBL5	0.669	UP_DR-REC	8.23 × 10^−5^	0.0299	0.0003	0.1433	0.0001	0.0381
28497-28	KPNA3	1.192	UP_DR-REC	0.0003	0.0408	0.0007	0.1598	0.0001	0.0399
11614-29	ERH	0.975	UP_DR-REC	0.0010	0.0694	0.0007	0.1598	0.0001	0.0425
8265-225	TMPO	1.922	UP_DR-REC	0.0003	0.0408	0.0008	0.1657	0.0001	0.0425
20137-49	HNRNPA1	1.073	UP_DR-REC	0.0006	0.0528	0.0003	0.1433	0.0001	0.0425
29827-184	LMNA	1.373	UP_DR-REC	0.0006	0.0528	0.0006	0.1598	0.0001	0.0464
20217-26	LMNB2	1.944	UP_DR-REC	8.23 × 10^−5^	0.0299	0.0007	0.1598	0.0002	0.0464
24943-117	FKBP15	1.658	UP_DR-REC	0.0002	0.0354	0.0008	0.1618	0.0002	0.0464
9831-12	G3BP2	1.726	UP_DR-REC	8.23 × 10^−5^	0.0299	0.0008	0.1618	0.0002	0.0464
2835-1	XRCC6	1.000	UP_DR-REC	0.0003	0.0408	0.0004	0.1528	0.0002	0.0471
5759-10	EFNA3	1.007	UP_DR-REC	0.0003	0.0408	0.0006	0.1598	0.0002	0.0471
15331-47	RBBP4	1.659	UP_DR-REC	0.0003	0.0408	0.0005	0.1598	0.0002	0.0496

All hits are directionally UP in serum obtained at clinically apparent recurrence (DR-REC) relative to diagnosis-timepoint serum from patients who later recurred (DR-DIG). log_2_FC: log_2_ fold-change (DR-REC vs. DR-DIG).

**Table 4:** Secretome-filtered candidate proteins passing the HPA-based filter: HPA Secretome and/or HPA Cancer proteome panel (S-BSST935), after exclusion of housekeeping genes and hematopoietic/blood-lineage-restricted genes.

Model	SeqId	Gene	log_2_FC	Direction	*p* (Limma)	FDR (Limma)	Uhlen 2019 Category	Uhlen “Secreted to Blood”	Alvez 2023 Plasma Panel
Monitor (DR-DIG vs. DR-REC)	29826-44	FUS	1.661	UP_DR-REC	8.84 × 10^−5^	0.0380	Not classified	No	Yes
Monitor (DR-DIG vs. DR-REC)	6036-78	EPHA10	1.739	UP_DR-REC	7.76 × 10^−5^	0.0380	Intracellular or membrane-bound	No	Yes
Monitor (DR-DIG vs. DR-REC)	5730-60	CXCL14	0.589	UP_DR-REC	7.45 × 10^−5^	0.0380	Secreted to blood	Yes	Yes
Monitor (DR-DIG vs. DR-REC)	6296-36	OIT3	1.428	UP_DR-REC	5.67 × 10^−5^	0.0380	Secreted to blood	Yes	No
Prediction (NR vs. DR-DIG)	29520-118	HPR	1.363	UP_DR-DIG	0.0010	0.9275	Secreted to blood	Yes	No
Prediction (NR vs. DR-DIG)	18397-5	AKR1C4	0.696	UP_DR-DIG	0.0135	0.9275	Not classified	No	Yes
Prediction (NR vs. DR-DIG)	12370-30	APOF	−0.680	UP_NR	0.0422	0.9275	Secreted to blood	Yes	No

Of 38 monitor hits, 4 passed (FUS, EPHA10, CXCL14, OIT3); of 25 nominal prediction hits, 3 passed (HPR, AKR1C4, APOF). NR: no recurrence; DR-DIG: diagnosis-timepoint serum from patients who later recurred; DR-REC: serum at clinically apparent recurrence.

**Table 5:** GO Biological Process, GO Molecular Function, and Reactome terms reaching Benjamini–Hochberg FDR < 0.05 by over-representation analysis (ORA; gseapy/Enrichr, Fisher’s exact test) of the 37 unique monitor-model hit gene symbols, tested against a background restricted to the 9482 genes measured on the SomaScan v5.0 panel (n = 22 terms, ranked by FDR).

Library	Term	n Overlap	Odds Ratio	FDR	Genes
GO Molecular Function 2023	RNA Binding (GO:0003723)	13	6.43	0.0002	CPSF7; XRCC6; FUS; SRSF1; HNRNPDL; PUF60; EWSR1; FUBP1; G3BP2; LONP1; ZNF207; ERH; HNRNPA1
Reactome 2022	mRNA Splicing R-HSA-72172	5	14.31	0.0063	CPSF7; PUF60; FUS; SRSF1; HNRNPA1
Reactome 2022	mRNA Splicing—Major Pathway R-HSA-72163	5	14.90	0.0063	CPSF7; PUF60; FUS; SRSF1; HNRNPA1
Reactome 2022	Processing Of Capped Intron-Containing Pre-mRNA R-HSA-72203	5	11.46	0.0115	CPSF7; PUF60; FUS; SRSF1; HNRNPA1
GO Molecular Function 2023	Single-Stranded DNA Binding (GO:0003697)	3	17.27	0.0325	FUBP1; LONP1; HNRNPA1
Reactome 2022	Depolymerisation Of Nuclear Lamina R-HSA-4419969	2	59.91	0.0371	LMNA; TMPO
Reactome 2022	Initiation Of Nuclear Envelope (NE) Reformation R-HSA-2995383	2	59.91	0.0371	LMNA; TMPO
GO Molecular Function 2023	Transcription Coactivator Binding (GO:0001223)	2	35.92	0.0421	STAT1; PGR
GO Molecular Function 2023	Single-Stranded RNA Binding (GO:0003727)	2	25.64	0.0463	LONP1; HNRNPA1
GO Molecular Function 2023	mRNA Binding (GO:0003729)	4	7.12	0.0463	CPSF7; FUBP1; SRSF1; G3BP2
GO Biological Process 2023	Nuclear Envelope Organization (GO:0006998)	2	77.04	0.0472	LMNA; LMNB2
GO Biological Process 2023	Nuclear Migration (GO:0007097)	2	77.04	0.0472	LMNA; LMNB2
GO Biological Process 2023	Regulation Of RNA Metabolic Process (GO:0051252)	3	18.43	0.0472	FUS; SRSF1; HNRNPA1
GO Biological Process 2023	Alternative mRNA Splicing, Via Spliceosome (GO:0000380)	2	49.01	0.0472	PUF60; SRSF1
GO Biological Process 2023	Protein Complex Oligomerization (GO:0051259)	3	15.64	0.0472	CPSF7; FUS; G3BP2
GO Biological Process 2023	Mitotic Spindle Checkpoint Signaling (GO:0071174)	2	38.49	0.0472	CDC20; ZNF207
GO Biological Process 2023	mRNA Splice Site Recognition (GO:0006376)	2	41.46	0.0472	PUF60; SRSF1
GO Biological Process 2023	Spindle Assembly Checkpoint Signaling (GO:0071173)	2	38.49	0.0472	CDC20; ZNF207
GO Biological Process 2023	Protein Localization To Nuclear Envelope (GO:0090435)	2	107.89	0.0472	LMNA; LMNB2
GO Biological Process 2023	Negative Regulation Of Mitotic Metaphase/Anaphase Transition (GO:0045841)	2	38.49	0.0472	CDC20; ZNF207
GO Biological Process 2023	Regulation Of RNA Splicing (GO:0043484)	3	15.64	0.0472	FUS; SRSF1; HNRNPA1
GO Biological Process 2023	Mitotic Spindle Assembly Checkpoint Signaling (GO:0007094)	2	38.49	0.0472	CDC20; ZNF207

**Table 6:** Elastic-net composite prediction-model panel (four genes selected by penalized Cox regression, l1 ratio = 0.5, fitted in the GSE103091 transcriptomic cohort using the candidate gene space nominated by the serum DEP analysis).

**(A) Panel composition and coefficients.**					
**Gene**		**Coefficient**		**DEP direction**	
ERAP1		−0.2414		UP_NR	
GZMA		−0.0663		UP_NR	
CLEC4C		−0.0297		UP_DR-DIG	
NRG3		−0.0134		UP_NR	
**(B) 103091. (n = 107 with metastasis-free survival annotation, 31 events).**					
**Model**	**l1 ratio**	**Nested-CV C-index**	**Nested-CV AUC (3-yr)**	**Nested-CV AUC (5-yr)**	**Median penalizer (λ)**
ElasticNet	0.50	0.613	0.657	0.638	0.32
LASSO	1.00	0.637	0.680	0.653	0.32
Naive z-sum		0.604	0.577	0.614	
**(C) External validation in GSE21653.**					
**External cohort**		**Endpoint**	**n**	**Events**	**C-index**
GSE21653_Basal		DFS	74	21	0.559

DFS: disease-free survival, AUC: area under the curve.

**Table 7:** Cox proportional-hazards HR, 95% CI, and *p*-values for prediction-model candidate genes across three independent GEO transcriptomic cohorts with survival annotation.

Cohort	Gene	DEP Direction	n	Events	HR (95% CI)	*p*
GSE103091 (MFS)	ERAP1	UP_NR	107	31	0.606 (0.432–0.852)	0.0039
GSE103091 (MFS)	GZMA	UP_NR	107	31	0.795 (0.657–0.961)	0.0177
GSE103091 (MFS)	CLEC4C	UP_DR-DIG	107	31	0.775 (0.603–0.996)	0.0464
GSE103091 (MFS)	CLEC12A	UP_NR	107	31	0.847 (0.700–1.024)	0.0860
GSE103091 (MFS)	CTSH	UP_DR-DIG	107	31	0.727 (0.501–1.055)	0.0937
GSE103091 (MFS)	NRG3	UP_NR	107	31	0.872 (0.738–1.030)	0.1066
GSE103091 (MFS)	DEFA6	UP_NR	107	31	1.240 (0.913–1.685)	0.1677
GSE103091 (MFS)	KLRG2	UP_NR	107	31	1.177 (0.930–1.490)	0.1739
GSE103091 (MFS)	STATH	UP_DR-DIG	107	31	1.138 (0.937–1.383)	0.1919
GSE103091 (MFS)	DEFA5	UP_NR	107	31	0.885 (0.713–1.098)	0.2668
GSE103091 (MFS)	PLAC1	UP_DR-DIG	107	31	1.099 (0.901–1.342)	0.3508
GSE103091 (MFS)	APOF	UP_NR	107	31	0.914 (0.754–1.107)	0.3575
GSE103091 (MFS)	ZFYVE27	UP_NR	107	31	1.373 (0.651–2.892)	0.4050
GSE103091 (MFS)	IFNGR1	UP_NR	107	31	0.788 (0.435–1.427)	0.4317
GSE103091 (MFS)	UGDH	UP_DR-DIG	107	31	1.163 (0.760–1.779)	0.4862
GSE103091 (MFS)	HPR	UP_DR-DIG	107	31	0.948 (0.760–1.181)	0.6319
GSE103091 (MFS)	AKR1C4	UP_DR-DIG	107	31	0.966 (0.739–1.262)	0.7985
GSE103091 (MFS)	PSMA4	UP_DR-DIG	107	31	0.923 (0.499–1.709)	0.7996
GSE103091 (MFS)	SIGLEC11	UP_NR	107	31	0.969 (0.745–1.261)	0.8153
GSE103091 (MFS)	PCDHGC3	UP_DR-DIG	107	31	1.031 (0.778–1.366)	0.8332
GSE103091 (MFS)	GSTM4	UP_NR	107	31	1.055 (0.584–1.904)	0.8593
GSE103091 (MFS)	ITCH	UP_NR	107	31	0.976 (0.554–1.719)	0.9315
GSE103091 (MFS)	CD177	UP_NR	107	31	1.007 (0.797–1.272)	0.9547
GSE21653 Basal (DFS)	GZMA	UP_NR	74	21	0.186 (0.057–0.614)	0.0057
GSE21653 Basal (DFS)	NRG3	UP_NR	74	21	0.183 (0.031–1.081)	0.0609
GSE21653 Basal (DFS)	CLEC4C	UP_DR-DIG	74	21	0.132 (0.013–1.362)	0.0890
GSE21653 Basal (DFS)	ERAP1	UP_NR	74	21	0.847 (0.135–5.290)	0.8585
GSE25066 Basal (DRFS)	ERAP1	UP_NR	189	65	2.910 (0.545–15.527)	0.2113
GSE25066 Basal (DRFS)	GZMA	UP_NR	189	65	1.131 (0.362–3.526)	0.8324

GSE103091 (n = 107, metastasis-free survival [MFS]), GSE21653 (n = 74, Basal-subtype, disease-free survival [DFS]), and GSE25066 (n = 189, Basal-subtype, distant recurrence-free survival [DRFS]). GZMA is the only candidate significant with concordant direction in two of three cohorts (GSE103091, GSE21653). NR: no recurrence; DR-DIG: diagnosis-timepoint serum from patients who later recurred.

## Data Availability

Original datasets used in the generation of this manuscript are available from the Corresponding Author, upon reasonable request.

## Supplementary Materials

The following supporting information can be downloaded at: https://www.mdpi.com/article/10.3390/ijms27209070/s1.

## Abbreviations

The following abbreviations are used in this manuscript:

TNBC	triple-negative breast cancer
DEP	differential expression protein
DEG	differentially expressed gene
FDR	false discovery rate
HPA	Human Protein Atlas
GEO	Gene Expression Omnibus
MFS	metastasis-free survival
DFS	disease-free survival
DRFS	distant recurrence-free survival
HR	hazard ratio
CI	confidence interval
ORA	over-representation analysis
GO	Gene Ontology
C-index	concordance index
