# Supplementary material for: Serum Proteomic Profiling by SomaScan Aptamer Array Identifies Candidate Predictive and Monitoring Biomarkers of Distant Recurrence in Triple-Negative Breast Cancer
Source: Int J Mol Sci. 2026 Oct 13;27(20):9070. doi: 10.3390/ijms27209070 (PMC13704957; doi:10.3390/ijms27209070)
Supplement: Supplementary file 1 [file ijms-27-09070-s001.zip › ijms-4569770-supplementary.pdf]

Supplementary Table S1. De novo Stage IV sensitivity analysis for all 38 limma-FDR monitor hits (DR-DIG vs DR-REC). Sensitivity comparison excludes the 4 de novo AJCC Stage IV samples from DR-REC (true DR-REC, n=5) vs DR-DIG (n=8); Mann–Whitney U test (appropriate given n=5). log2FC = mean(true DR-REC) - mean(DR-DIG); + = higher at recurrence, consistent with the primary UP_DR-REC direction for all 38 hits. Perfect separation = all 5 true-DR-REC values exceed all 8 DR-DIG values.

**Gene**	**SeqId**	**Primary log2FC**	**Primary p (limma)**	**Primary FDR**	**Sensitivity log2FC**	**Sensitivity p (MWU, n=5 vs 8)**	**Nominal sig. (p<0.05)**	**Perfect separation**
DPY30	13943-38	2.4018	7.82e-06	0.0361	2.1593	0.0016	Yes	Yes
FUBP1	20960-47	1.4699	1.07e-05	0.0361	1.5171	0.0016	Yes	Yes
DCDC2	31440-109	1.2865	1.43e-05	0.0361	1.3006	0.0016	Yes	Yes
BAGE2	6294-11	1.7289	1.55e-05	0.0361	1.6749	0.0031	Yes	No
HNRNPDL	10852-114	1.2134	1.72e-05	0.0361	1.4640	0.0016	Yes	Yes
ABHD14A	5715-4	1.5932	2.83e-05	0.0361	1.5242	0.0031	Yes	No
ZNF394	33622-23	1.3470	2.84e-05	0.0361	1.3011	0.0016	Yes	Yes
SPIRE2	31035-50	1.0594	3.26e-05	0.0361	1.0316	0.0016	Yes	Yes
PGR	15693-9	1.4810	3.87e-05	0.0361	1.5517	0.0016	Yes	Yes
ZNF207	25814-34	1.6080	4.18e-05	0.0361	1.5509	0.0016	Yes	Yes
SRSF1	26209-2	1.6550	4.40e-05	0.0361	1.5806	0.0031	Yes	No
LY6G6C	6256-9	1.2154	4.43e-05	0.0361	1.1608	0.0109	Yes	No
CDC20	28494-11	1.0905	4.45e-05	0.0361	1.0627	0.0109	Yes	No
LONP1	6398-12	1.3435	4.78e-05	0.0361	1.4019	0.0062	Yes	No
OIT3	6296-36	1.4281	5.67e-05	0.0380	1.3205	0.0062	Yes	No
CLSTN3	6291-55	1.6323	6.20e-05	0.0380	1.5000	0.0016	Yes	Yes
CXCL14	5730-60	0.5885	7.45e-05	0.0380	0.6052	0.0016	Yes	Yes
STAT1	10370-21	0.7742	7.61e-05	0.0380	0.7151	0.0031	Yes	No
EPHA10	6036-78	1.7387	7.76e-05	0.0380	1.5697	0.0062	Yes	No
CPSF7	29942-8	0.9230	7.78e-05	0.0380	0.8949	0.0031	Yes	No
EWSR1	12988-49	1.4691	8.76e-05	0.0380	1.3843	0.0016	Yes	Yes
FUS	29826-44	1.6615	8.84e-05	0.0380	1.6343	0.0031	Yes	No
FAM161A	33227-149	1.4930	8.92e-05	0.0380	1.5363	0.0062	Yes	No
PUF60	10575-31	1.7164	9.01e-05	0.0380	1.4994	0.0016	Yes	Yes
HNRNPA1	12466-7	0.9520	9.65e-05	0.0381	1.0417	0.0062	Yes	No
UTS2B	6290-3	0.5513	1.00e-04	0.0381	0.5454	0.0062	Yes	No
UBL5	21104-37	0.6686	1.01e-04	0.0381	0.7005	0.0016	Yes	Yes
KPNA3	28497-28	1.1915	1.10e-04	0.0399	1.1703	0.0062	Yes	No
ERH	11614-29	0.9746	1.24e-04	0.0425	0.8045	0.0186	Yes	No
TMPO	8265-225	1.9219	1.28e-04	0.0425	1.7536	0.0062	Yes	No
HNRNPA1	20137-49	1.0730	1.29e-04	0.0425	1.1449	0.0109	Yes	No
LMNA	29827-184	1.3730	1.46e-04	0.0464	1.0084	0.0109	Yes	No
LMNB2	20217-26	1.9442	1.50e-04	0.0464	1.8066	0.0016	Yes	Yes
FKBP15	24943-117	1.6579	1.56e-04	0.0464	1.5839	0.0031	Yes	No
G3BP2	9831-12	1.7259	1.58e-04	0.0464	1.7193	0.0016	Yes	Yes
XRCC6	2835-1	0.9998	1.69e-04	0.0471	0.9136	0.0062	Yes	No
EFNA3	5759-10	1.0074	1.70e-04	0.0471	1.3016	0.0031	Yes	No
RBBP4	15331-47	1.6585	1.83e-04	0.0496	1.6766	0.0062	Yes	No

Summary: 38/38 genes (100%) remain nominally significant (p<0.05) at n=5 vs 8; 15/38 genes (39.5%) show perfect rank separation. All 38 genes were directly re-analyzed (superseding the earlier partial 33/38 estimate, which was computed on the pre-rebuild 81-gene MWU hit list overlap).

Supplementary Table S2. Clinical characteristics at the time of serum collection in the analyzed TNBC groups (number of patients). Data were obtained by review of the medical records by the BORN-Biobank investigators. Patients may appear in more than one comorbidity or medication category. One patient contributed both a DR-DIG and a DR-REC sample and is counted in both columns.

Characteristic	NR (n=12)	DR-DIG (n=8)	DR-REC (n=9)
**Timing of serum collection**			
At diagnosis, before any treatment	12	8	4 (de novo Stage IV)
At diagnosis of distant recurrence, before treatment for recurrent disease	0	0	5
**Status at collection**			
Active infection	0	0	0
Hepatic or renal dysfunction	0	0	0
**Comorbidities and medical history**			
Cardiovascular or cerebrovascular disease, or hypertension	2	2	0
Dyslipidemia	0	0	1
Myelodysplastic syndrome	0	0	1
Prior non-breast malignancy (treated, no recurrence)	1	1	0
Status post adrenal tumor resection	0	1	0
Other (glaucoma or suspected glaucoma, Ménière's disease, osteoarthritis, peripheral circulatory disorder, prior myomectomy)	2	3	1
None recorded	7	2	6
Not reported	1	0	0
**Concomitant medications**			
Antiplatelet agent	1	1	0
Statin	2	1	0
Antihypertensive or antianginal agent	4	2	0
Corticosteroid (hydrocortisone replacement)	0	1	0
Thyroid hormone (levothyroxine)	0	0	1
Non-steroidal anti-inflammatory drug	0	0	2
Opioid	0	0	1
Hypnotic or anxiolytic (including melatonin)	2	1	3
Gastrointestinal or antiemetic agent	1	1	4
Other (vitamin E, bisphosphonate, betahistine, adenosine triphosphate, camostat, dietary supplement)	2	1	2
None	5	5	3
Not reported	0	0	1
**Serum storage**			
Storage duration, years, median (range)	8.1 (2.7–13.4)	5.0 (3.5–12.4)	10.0 (1.0–13.6)

Hepatic or renal dysfunction: no Grade ≥2 hepatic or renal laboratory abnormality recorded at or near the time of collection. Storage duration: from serum collection to release from the biobank; all samples were released on the same date. The time from collection to processing and freezing, freeze–thaw history, and hemolysis status were not recorded. NR: no recurrence; DR-DIG: diagnosis-timepoint serum from patients who later developed distant recurrence; DR-REC: serum at clinically apparent recurrence or de novo Stage IV presentation.

Supplementary Table S3. Leave-one-out analysis of the 38 monitor-model hits (DR-DIG, n=8, vs DR-REC). Each DR-REC sample was excluded in turn, and each aptamer was re-tested by two-sided Mann–Whitney U test.

Excluded sample	DR-REC n	Hits with p<0.05	Hits higher in DR-REC	Median log2FC	Largest p
None (all 9 DR-REC samples)	9	38/38	38/38	1.40	0.0010
DR-REC sample 1 —distant recurrence	8	38/38	38/38	1.51	0.0019
DR-REC sample 2 —distant recurrence (myelodysplastic syndrome)	8	38/38	38/38	1.39	0.0019
DR-REC sample 3 —distant recurrence	8	38/38	38/38	1.27	0.0019
DR-REC sample 4 —distant recurrence	8	38/38	38/38	1.51	0.0011
DR-REC sample 5 —distant recurrence	8	38/38	38/38	1.47	0.0019
DR-REC sample 6 —de novo Stage IV	8	38/38	38/38	1.42	0.0019
DR-REC sample 7 —de novo Stage IV	8	38/38	38/38	1.31	0.0019
DR-REC sample 8 —de novo Stage IV	8	38/38	38/38	1.39	0.0019
DR-REC sample 9 —de novo Stage IV	8	38/38	38/38	1.44	0.0019

log2FC: log2 fold-change (DR-REC vs DR-DIG). Samples are numbered for this table only.

Supplementary Table S4. Serum storage duration and the 38 monitor-model hits. (A) Spearman correlation between the per-sample signature score (mean z-score of the 38 aptamers relative to DR-DIG) and storage duration. (B) Linear regression of log2 abundance on group (DR-REC vs DR-DIG, n=9 vs 8) with and without storage duration (years) as a covariate. Storage duration by group is given in Supplementary Table S2 (DR-DIG vs DR-REC, Mann–Whitney U p=0.42).

(A) Signature score vs storage duration.

**Samples**	**Spearman ρ**	**p**
DR-DIG+NR (n=20)	0.18	0.458
DR-DIG (n=8)	0.29	0.493
NR (n=12)	0.07	0.829
DR-REC (n=9)	0.33	0.381

(B) Storage-adjusted group effect for each aptamer.

**Gene**	**SeqId**	**Group β (unadjusted)**	**Group β (storage-adjusted)**	**Group p (adjusted)**	**Attenuation (%)**	**Storage β per year**	**Storage p**
DCDC2	31440-109	1.287	1.148	3.89e-05	10.8	0.064	0.020
DPY30	13943-38	2.402	2.248	8.15e-05	6.4	0.071	0.190
FUBP1	20960-47	1.470	1.373	8.33e-05	6.6	0.045	0.178
ZNF394	33622-23	1.347	1.197	9.36e-05	11.1	0.069	0.026
SPIRE2	31035-50	1.059	0.926	1.01e-04	12.6	0.061	0.013
HNRNPDL	10852-114	1.213	1.196	1.13e-04	1.4	0.008	0.779
BAGE2	6294-11	1.729	1.605	1.32e-04	7.1	0.057	0.162
ABHD14A	5715-4	1.593	1.431	1.38e-04	10.2	0.075	0.048
CDC20	28494-11	1.090	0.962	1.58e-04	11.7	0.059	0.025
LY6G6C	6256-9	1.215	1.084	1.64e-04	10.8	0.060	0.040
ZNF207	25814-34	1.608	1.444	2.34e-04	10.2	0.076	0.060
SRSF1	26209-2	1.655	1.488	2.45e-04	10.1	0.077	0.063
ERH	11614-29	0.975	0.840	2.59e-04	13.8	0.062	0.013
PGR	15693-9	1.481	1.369	2.63e-04	7.6	0.052	0.165
UBL5	21104-37	0.669	0.667	3.21e-04	0.2	0.001	0.977
STAT1	10370-21	0.774	0.690	3.43e-04	10.9	0.039	0.052
OIT3	6296-36	1.428	1.288	3.84e-04	9.8	0.065	0.084
CXCL14	5730-60	0.589	0.556	3.86e-04	5.5	0.015	0.342
CLSTN3	6291-55	1.632	1.472	3.94e-04	9.8	0.074	0.086
LONP1	6398-12	1.343	1.314	3.99e-04	2.2	0.014	0.705
CPSF7	29942-8	0.923	0.833	4.40e-04	9.7	0.041	0.091
UTS2B	6290-3	0.551	0.513	4.44e-04	7.0	0.018	0.224
EPHA10	6036-78	1.739	1.571	5.10e-04	9.6	0.077	0.100
HNRNPA1	12466-7	0.952	0.869	5.18e-04	8.7	0.038	0.137
KPNA3	28497-28	1.192	1.055	5.36e-04	11.4	0.063	0.052
FAM161A	33227-149	1.493	1.364	5.54e-04	8.6	0.059	0.145
EWSR1	12988-49	1.469	1.337	5.88e-04	9.0	0.061	0.130
TMPO	8265-225	1.922	1.681	6.06e-04	12.5	0.111	0.036
FUS	29826-44	1.661	1.549	6.53e-04	6.8	0.052	0.263
PUF60	10575-31	1.716	1.594	6.58e-04	7.2	0.057	0.237
LMNB2	20217-26	1.944	1.694	6.82e-04	12.9	0.116	0.033
EFNA3	5759-10	1.007	0.988	8.39e-04	2.0	0.009	0.761
HNRNPA1	20137-49	1.073	0.987	8.52e-04	8.0	0.040	0.198
FKBP15	24943-117	1.658	1.476	8.58e-04	11.0	0.084	0.077
XRCC6	2835-1	1.000	0.896	9.90e-04	10.4	0.048	0.100
LMNA	29827-184	1.373	1.305	1.03e-03	5.0	0.031	0.442
G3BP2	9831-12	1.726	1.649	1.09e-03	4.5	0.036	0.491
RBBP4	15331-47	1.659	1.542	1.25e-03	7.0	0.054	0.280

All 38 aptamers remained higher in DR-REC after adjustment (group $p<0.05$ for all; median attenuation 9%, maximum 14%). 9 of 38 aptamers showed a nominal association with storage duration ($p<0.05$). In a further check restricted to the 5 DR-REC samples whose storage duration fell within the DR-DIG range, all 38 aptamers were higher in DR-REC with $p<0.05$ (Mann–Whitney U). Group and storage duration are partly collinear; these analyses are exploratory. β: regression coefficient on the log2 scale.
